# Transcriptome-Based Analysis of Dof Family Transcription Factors and Their Responses to Abiotic Stress in Tea Plant (*Camellia sinensis*)

**DOI:** 10.1155/2016/5614142

**Published:** 2016-10-31

**Authors:** Hui Li, Wei Huang, Zhi-Wei Liu, Yong-Xin Wang, Jing Zhuang

**Affiliations:** ^1^Tea Science Research Institute, College of Horticulture, Nanjing Agricultural University, Nanjing 210095, China; ^2^State Key Laboratory of Crop Genetics and Germplasm Enhancement, College of Horticulture, Nanjing Agricultural University, Nanjing 210095, China

## Abstract

Tea plant (*Camellia sinensis* (L.) O. Kuntze) is affected by abiotic stress during its growth and development. DNA-binding with one finger (Dof) transcription factors (TFs) play important roles in abiotic stress tolerance of plants. In this study, a total of 29 putative Dof TFs were identified based on transcriptome of tea plant, and the conserved domains and common motifs of these CsDof TFs were predicted and analyzed. The 29 CsDof proteins were divided into 7 groups (A, B1, B2, C1, C2.1, C2.2, and D2), and the interaction networks of Dof proteins in* C. sinensis* were established according to the data in* Arabidopsis*. Gene expression was analyzed in “Yingshuang” and “Huangjinya” under four experimental stresses by qRT-PCR.* CsDof* genes were expressed differentially and related to different abiotic stress conditions. In total, our results might suggest that there is a potential relationship between CsDof factors and tea plant stress resistance.

## 1. Introduction

Transcription factors (TFs), also known as* trans*-acting factors, are proteins that recognize and bind specific DNA sequences. These TFs can not only participate in various biological processes to activate or repress plant metabolic [1], but also act as regulators in the process of stem elongation and seed development [2]. Furthermore, some TFs could also increase plant resistance and grain yield [3, 4].

DNA-binding with one finger (Dof) family is a transcription factor family in plants [5]. The first* Dof* gene was found in maize [6]. Dof TFs play a role in transcriptional regulation, which is characterized by an N-terminal highly conserved DNA-binding domain and a C-terminal domain [6–8]. Additionally, Dof TFs generally comprise 200–400 amino acid residues with a Dof domain which functions as a Cys2/Cys2 zinc finger domain [9]. Dof TFs regulate the expression of genes involved in plant development and defense processes, such as seed maturation and germination [10–12], plant defense mechanisms [13], photoperiodic flowering time [14, 15], and secondary metabolism [16].

In higher plant, there were various amounts of Dof TFs. Based on genome or transcriptome sequences, a total of 20, 36, 38, 42, 76, and 78 Dof TFs have been identified in* Chrysanthemum* [17],* Arabidopsis* [18], pigeon pea [19],* Medicago truncatula *[20], Chinese cabbage [21], and soybean [22], respectively. Some members of Dof TF family have been extensively studied from a variety of plant species. AtDOF4.7 has been identified to play a role in floral organ abscission in* Arabidopsis* [23]. PpDof1 serves as transcriptional repressor and is involved in the growth of nutrient-dependent filament in* Physcomitrella patens* [24].* BnCDF1*, a member of Dof family TF, takes part in regulating the flowering time and freezing tolerance in* Brassica napus* [25]. Overexpression of* PpDof5 *gene from maritime pine in transgenic* Arabidopsis* enhances the content of lignin [26]. The transgenic rice plants hosting* OsDof12* gene exhibit a change of plant architecture [27]. Meanwhile, the expression of* CaDofs* in response to abiotic stress was observed in pepper [28].

Tea plant (*Camellia sinensis* (L.) O. Kuntze) is native to East Asia, which is probably originated from the northern part of the Burma, Yunnan, and Sichuan of China [29]. Tea plant is an important commercially valuable economic crop and has been cultivated for at least 2000 years in China [30]. Tea is an aromatic beverage and usually made of the leaves of tea plant. Moreover, numerous researches have revealed that tea is helpful to human health. Green tea could reduce the risk of cardiac injury following ischemia because of its excellent source of antioxidants [31]. Additionally, the extracts of tea could be used as medicine for treatment of neurodegenerative diseases [32], a neuroprotective agent for Parkinson's disease [33]. The leaves of “Yingshaung” are green. “Huangjinya” is a spontaneous mutant, and its leaves are pale yellow. Plants growth and production are greatly affected by abiotic stress conditions (i.e., high/low temperatures, high salinity, and drought). The regulation of Dof TFs in response to abiotic stress has been investigated in other plants. Some researchers have also analyzed other TFs families in tea plant [34, 35]. There is, however, the regulatory mechanism of Dof TFs in tea plant which is not completely understood till now.

Our work aimed to investigate the Dof TFs in tea plant. In this work, a total of 29* CsDof* genes were identified from the transcriptome database of tea plant. Then, the phylogenetic relationships and motifs were also analyzed. Additionally, quantitative real-time PCR (qRT-PCR) was performed to detect the expression profiles of* CsDof* genes in two cultivars of tea plant (“Yingshuang” and “Huangjinya”) under four abiotic stress treatments (salt, drought, 38°C, and 4°C). Our results extensively studied Dof TFs in tea plant. The results provide insights into CsDof TFs in tea plant and offer useful resource to improve the resistance to abiotic stress in tea plant.

## 2. Materials and Methods

### 2.1. Materials

*C. sinensis* cvs. “Yingshuang” and “Huangjinya” were cutting seedlings from 5-year-old tea plants and cultivated at Nanjing Agriculture University, Nanjing, China (32°02′N, 118°50′E, altitude 20 m above mean sea level) in December 2014. The plants were grown in a chamber in a mixture of vermiculite and organic (1 : 1; v/v) and acidic soil (pH 5.6) at 25°C. The plants were exposed to stress treatments (4°C and 38°C temperature, salt, and drought), and then tea plant leaves were harvested at three treatments time points (0, 2, 8, and 24 h). All specimens were selected from the visible maturity of tea plant leaves and immediately frozen in liquid nitrogen and stored at −80°C before experimental analysis.

### 2.2. Methods

#### 2.2.1. Total RNA Isolation and cDNA Synthesis

Total RNA of the specimens was isolated using a commercial RNA extraction kit (Huayueyang, Beijing, China) in accordance with the manufacturer's instructions, and then the RNA concentration was measured immediately using a Nanodrop 2000 spectrophotometer (Thermo Scientific, Wilmington, DE). Complementary DNA (cDNA) libraries were constructed by using a PrimeScript RT reagent kit in accordance with the manufacturer's protocols (TaKaRa, Dalian, China). Then, the suitable cDNA fragments were selected as templates and eventually diluted 20-fold for qRT-PCR analysis after agarose gel electrophoresis filtration.

#### 2.2.2. Database Search of Dof TFs from Tea Plant

The amino acid sequences of Dof proteins in* Arabidopsis* were downloaded from Plant Transcription Factor Database (PlantTFDB) v3.0 (http://planttfdb.cbi.pku.edu.cn/) [36]. The sequences of* Dof* genes of tea plant were searched from the annotation information of* C. sinensis *transcriptome [37].

#### 2.2.3. Sequence Analysis and Phylogenetic Tree Construction

The conserved motifs of Dof factor sequences were identified using MEME (version 4.10.2) (http://meme-suite.org/) [38]. The parameters of MEME suite were set as follows: maximum number of motifs was 30; the other options of parameter were the default. The open reading frames (ORFs) and the translation of sequences were analyzed using BioXM version 2.6 (the Conserved Domains of sequences were identified using Blast https://blast.ncbi.nlm.nih.gov/Blast.cgi). The predicted protein-protein interactions were constructed by using STRING (version 10.0) (http://string-db.org/) [39]. The database of* A. thaliana* was selected for organism of STRING. The heat map was illustrated by using HemI 1.0 software (http://hemi.biocuckoo.org/faq.php) [40]. DNAMAN version 6.0 was performed to analyze the sequence alignments between the Dof TFs in* Arabidopsis* and* C. sinensis*. The phylogenetic tree was analyzed and constructed by using the bio-software MEGA version 5.0 and Clustal W [41, 42].

#### 2.2.4. qRT-PCR Analysis

A total of 8 genes were selected from* Dof* family of tea plant, and then specific primers of these genes for qRT-PCR were designed using Primer Premier version 6 (Table 1). All primers were synthesized in Genscript Nanjing Inc (Nanjing, China). qRT-PCR was conducted in real-time PCR platform Bio-Rad iQ5 (Bio-Rad Laboratories, Inc., Hercules, CA, USA). The reaction volume of qRT-PCR was 20 *μ*L with 2 *μ*L of a diluted cDNA sample as the template, 10 *μ*L of SYBR Premix* Ex Taq* (TaKaRa, Dalian, China), 7.2 *μ*L of deionized water, and 0.4 *μ*L of each gene-specific primer. The thermal cycling conditions of qRT-PCR were as follows: 95°C for 30 s; followed by 40 cycles at 95°C for 5 s and 60°C for 30 s; and then 61 cycles at 65°C for 10 min.* Csactin* was used to normalize the expression levels of* CsDof*s (*Csactin *forward primer: 5′-GATTCCGTTGCCCTGAAGTCCT-3′,* Csactin* reverse primer: 5′-CCTTGCTCATACGGTCTGCGATA-3′). Relative gene expression was calculated as 2^−ΔΔCT^ accordance with Pfaffl method [43].

#### 2.2.5. Statistical Analysis

The mean value was calculated on the basis of three technical replicates. Differences in expression levels were determined* via* Duncan's multiple-range test at a 0.05 probability level in SPSS17.0 (SPSS Inc., Chicago, IL, USA).

## 3. Results

### 3.1. Identification and Analysis of CsDof TFs

A total of 29 putative* CsDof* genes have been found on the basis of our transcriptome database of tea plants and were numbered from CsDof1 to CsDof29 (Table  S1 and Table  S2 in Supplementary Material available online at http://dx.doi.org/10.1155/2016/5614142). Then, the conserved domain was identified in* CsDof* genes with the analysis of NCBI Blast program.

### 3.2. Phylogenetic and Classification Analysis of Dof TFs in* C. sinensis*

**Figure 1:**
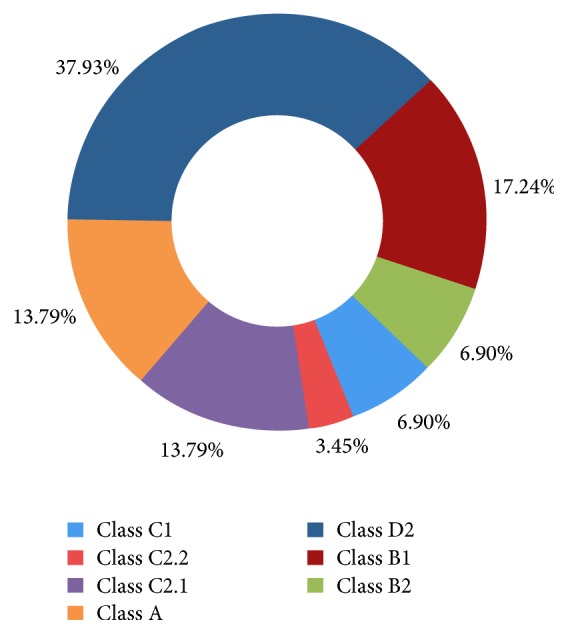
The proportions of various CsDof classes in* C. sinensis*.

**Figure 2:**
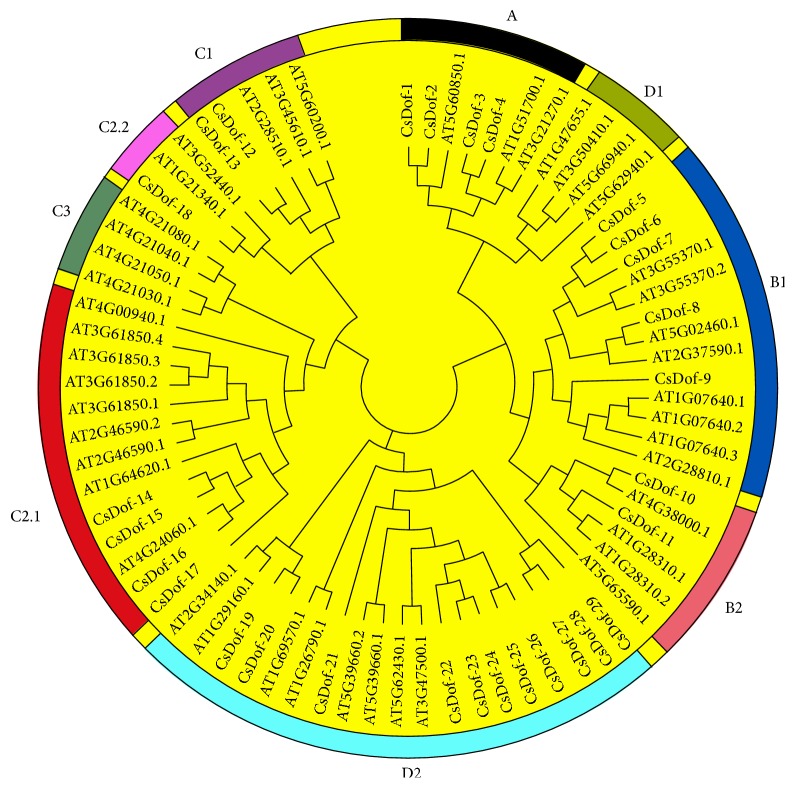
Unrooted phylogenetic tree of CsDofs in* C. sinensis*.

A total of 45 amino acid sequences of Dof TFs in* Arabidopsis* were downloaded from PlnTFDB (Table S3). The phylogenetic tree was constructed with 29 CsDof TFs in tea plant and 45 AtDof TFs in* Arabidopsis*. The proportion of each Dof subgroup of tea plant was constructed. There was no CsDof factor in Classes D1 and C3. The largest number of CsDof factors was Class D2, which showed the proportions of 37.93%, and the smallest class was Class C2.2, which shared proportion of 3.45%. Furthermore, Classes A and C2.1 shared same proportion of 13.79%. Classes C1 and B2 also shared individual proportion of 6.90% (Figure 1). These Dof TFs from tea plant and* Arabidopsis* were divided into 9 subgroups (A, B1, B2, C1, C2.1, C2.2, C3, D1, and D2) on the basis of the classification of Lijavetzky [18] (Figure 2).

### 3.3. Conserved Domain Discovery of CsDof TFs

**Figure 3:**
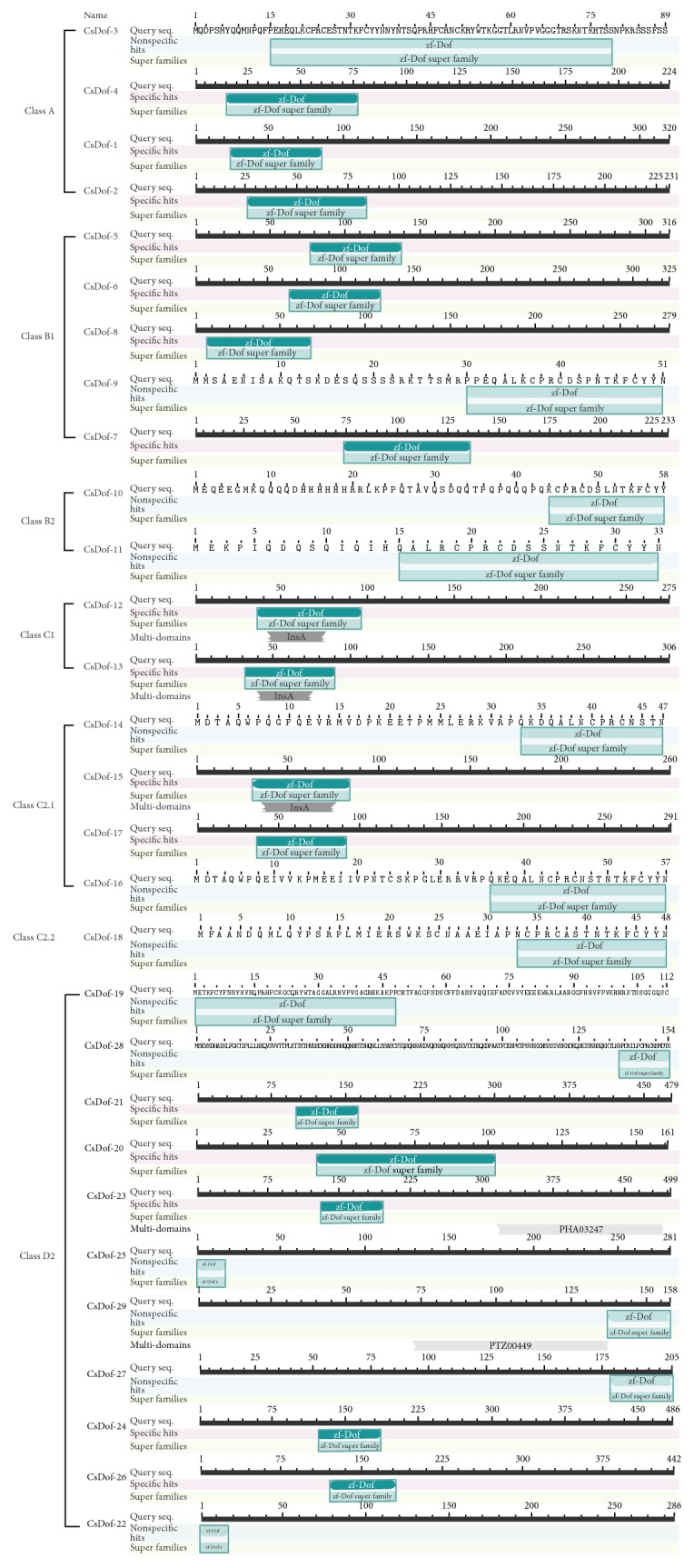
Conserved domains of CsDofs in* C. sinensis*.

Dof TFs were characterized by the highly conserved zf-Dof domain (DNA-binding domain with a single zing finger). The putative conserved domains of Dof TFs of tea plant have been detected by using Blast. The 29 CsDof family proteins had a highly conserved zf-Dof domain which showed resemblance to the Cys2 zinc finger and was significantly related to the N-terminal region. The 29 CsDof family proteins belonged to the zf-Dof super family (Figure 3). Some TFs were classified into the same class that had a similar zf-Dof domain. For instance, CsDof-15 and CsDof-17 had the similar site of conserved domain, and CsDof-25 and CsDof-22 had the similar site of conserved domain. All of the 29 CsDof family proteins had various confidence levels, a total of 13 CsDof family proteins had nonspecific hits, and 16 CsDof family proteins had specific hits.

### 3.4. Motif Discovery of CsDof TFs

**Figure 4:**
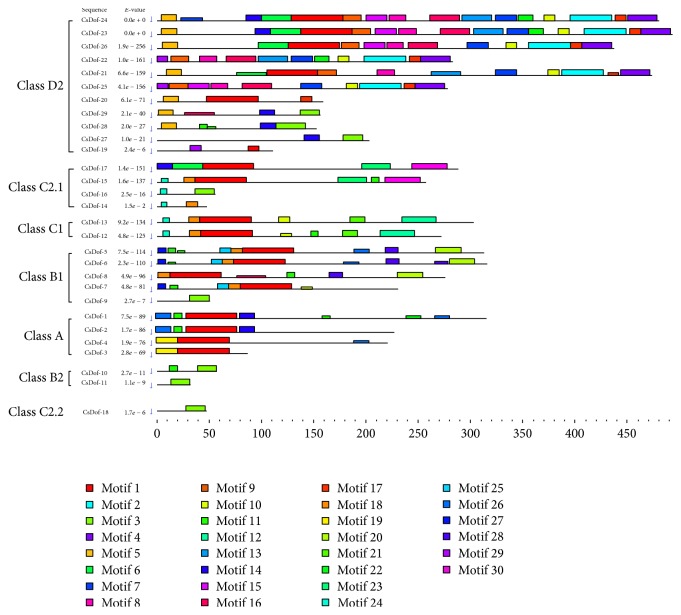
Common motifs of CsDof family proteins in* C. sinensis*.

**Figure 5:**
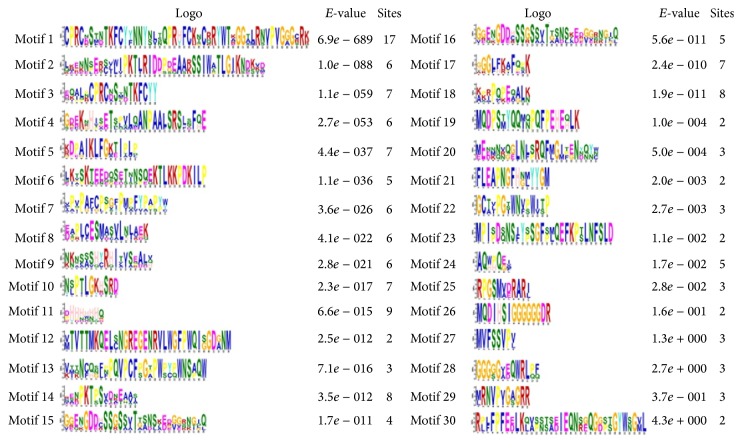
Sequence logos of Dof domains in* C. sinensis*. The overall height of the stack indicates the level of sequence conservation. Heights of residues within a stack indicate the relative frequency of each residue at that position.

The specific motifs of CsDof TFs were indicated by MEME program, and a total of 30 motifs were found from* C. sinensis* (Figures 4 and 5). Each of these CsDof TFs had an* E*-value less than 10. CsDof-24 contained 16 motifs, which contained the largest number of motifs. Only one motif was contained in CsDof-18, CsDof-9, and CsDof-11, respectively. Most of CsDof TFs contained motif 1 in the Dof domain region. The motifs in Class C2.1 were similar to that in Class C1. Motif 4, motif 5, and motif 17 only existed in Class D2.

### 3.5. Evolution of the Dof TFs Family among Plants

**Figure 6:**
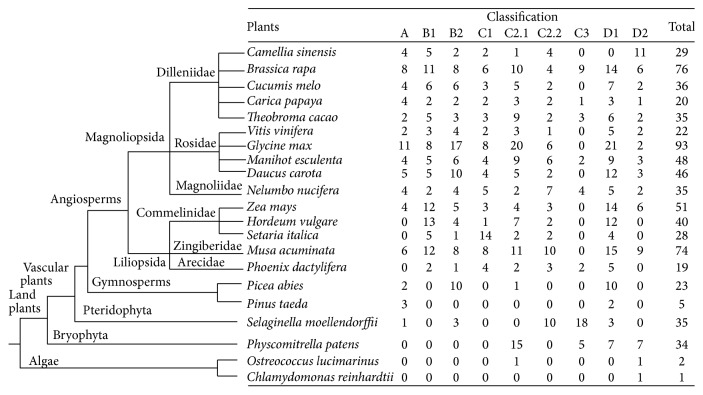
Comparisons of Dof TFs in different species.

Dof TFs among other species have been identified. In order to analyze the relationship between* C. sinensis* and other plants, the evolution of the Dof TFs in* C. sinensis* and other plants was constructed (Figure 6). Dof TFs in 21 species were compared, including* C. sinensis*. There was a notable difference among different species in the classification of Dof TFs. Moreover, the number of Dof TFs in land plant was higher than that in algae.

### 3.6. The Interaction Network of Dof TFs between* C. sinensis *and* Arabidopsis*

**Figure 7:**
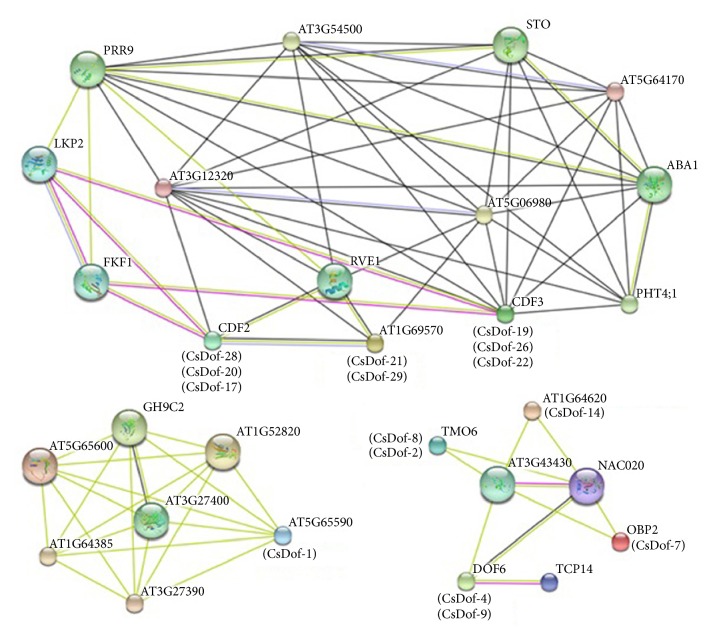
The interaction networks of Dofs in* C. sinensis* according to the orthologs in* Arabidopsis.*

The predicted protein-protein interactions were constructed associated with* Arabidopsis* by using the amino acid sequences of CsDof TFs from* C. sinensis* (Figure 7). Different line colors represent the types of evidence for the association. Similar proteins of CDF2 (Cycling Dof factor 2) and AT1G69570 played a role in regulating a photoperiodic flowering response, as well as CDF3 (Cycling Dof factor 3) in* A. thaliana*. The amino acid sequence of CDF2 showed a high similarity to the three CsDof TFs (CsDof-28, CsDof-20, and CsDof-17). AT1G69570 showed a high similarity to CsDof-21 and CsDof-29. AT5G65590 bound specifically to a 5′-AA[AG]G-3′ consensus core sequence. In addition, NAC020 was a domain containing protein. There was a complicated interaction between NAC020 and six CsDof TFs (CsDof-8, CsDof-2, CsDof-4, CsDof-9, CsDof-14, and CsDof-7).

### 3.7. Expression Profiles of* CsDof* Genes in Four Tea Plant Cultivars

**Figure 8:**
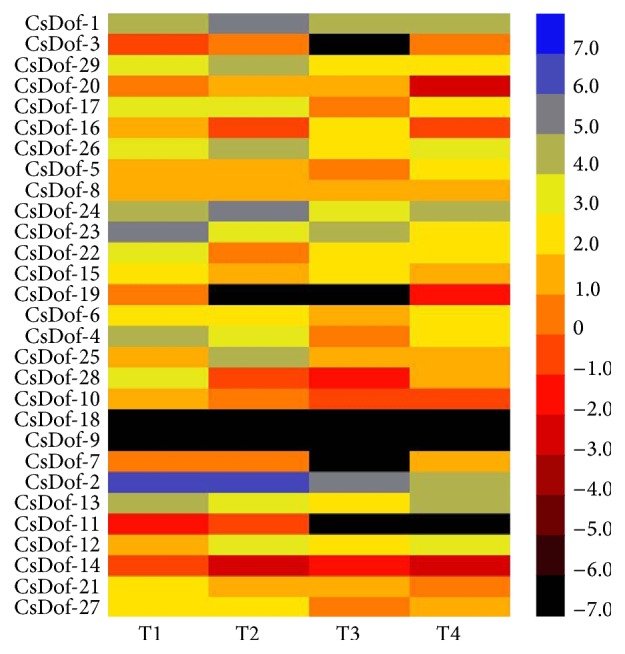
Heat map of Dof genes in four tea plant cultivars. T1 represents “Yunnanshilixiang,” T2 represents “Chawansanhao,” T3 represents “Ruchengmaoyecha,” and T4 represents “Anjibaicha.” Color scores were normalized by the log_2_ transformed counts of RPKM values.

RNA-seq data was extracted from transcriptome database and used to visualize expression information of 29* CsDof* genes in four tea plant cultivars [37], and then the heat map was constructed (Figure 8). RPKM (reads per kilobase per million mapped reads) values of 29* CsDof* genes were analyzed in the four cultivars, including “Yunnanshilixiang,” “Chawansanhao,” “Ruchengmaoyecha,” and “Anjibaicha.” T1 represents “Yunnanshilixiang,” T2 represents “Chawansanhao,” T3 represents “Ruchengmaoyecha,” and T4 represents “Anjibaicha”. Blue represents high expression level, and black represents slight expression level. Notably,* CsDof-9* and* CsDof-18* were not detected in the four tea plant cultivars. Most of the* CsDof* genes were more highly expressed in T1 and T2 than that in the other two cultivars (T3 and T4).* CsDof-2* showed the highest RPKM value (111.53) in T2. The expression levels of same genes were similar in different cultivars. For instance,* CsDof-7* was expressed similarly in T1 and T2, as well as* CsDof-17* and* CsDof-5*. Furthermore, different genes showed a similar expression level in the same cultivar were also observed.

### 3.8. Expression Analysis of* CsDof* Genes under Stress Treatments in Two* C. sinensis *Cultivars

To clarify the potential functions of* CsDof* genes in response to different abiotic stress treatments, the expression patterns of eight* CsDof* genes were analyzed through qRT-PCR under four abiotic stress conditions in “Yingshuang” and “Huangjinya.” Eight* CsDof* genes included* CsDof-10*,* CsDof-16*,* CsDof-8*,* CsDof-22*,* CsDof-9*,* CsDof-7*,* CsDof-2*, and* CsDof-13*. The abiotic stress treatments included high temperature (38°C), low temperature (4°C), high salt concentration (0.2 M NaCl), and drought stress treatment (200 g·L^−1^ PEG).

#### 3.8.1. High Temperature (38°C) Treatment

**Figure 9:**
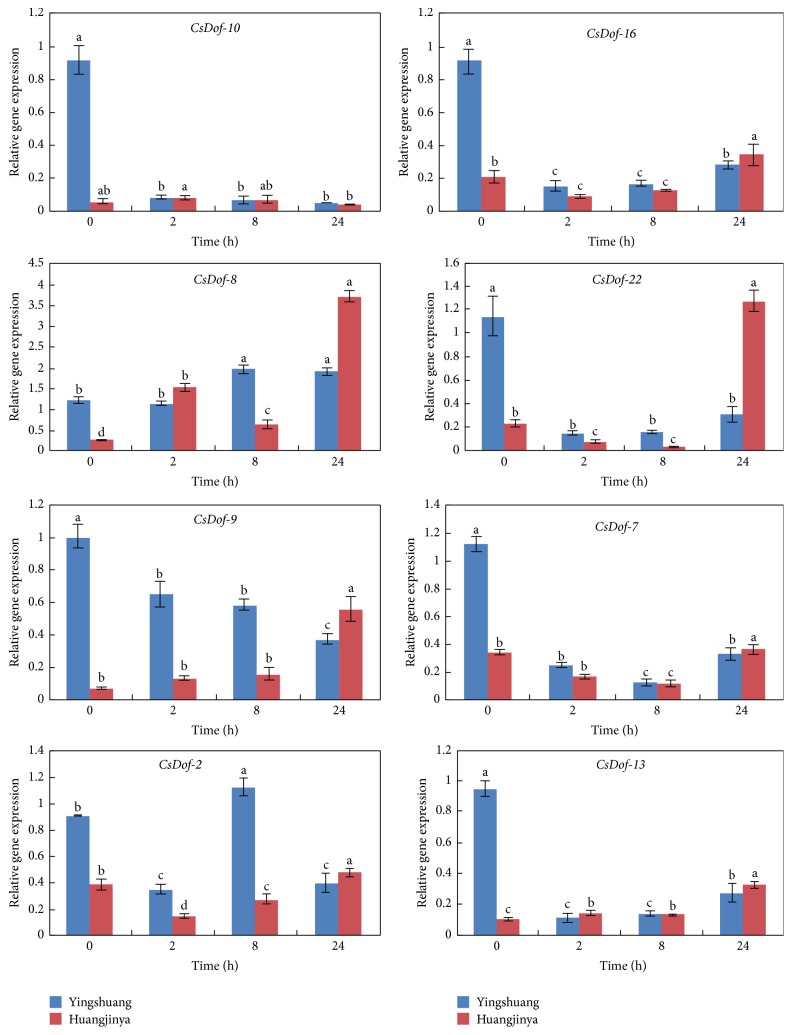
Expression patterns of* CsDof* genes in “Yingshuang” and “Huangjinya” under high temperature stress. Error bars represent standard deviation among three real-time quantitative PCR reaction replicates. Data are means of three technical replicates ± SD. Different lowercase letters indicate significant differences at *P* < 0.05.

Thetranscript levels of most of the selected* CsDof* genes were significantly decreasing under high temperature treatment in “Yingshuang.” However,* CsDof-8* gene increased firstly and then peaked at 8 h in “Yingshuang.”* CsDof-2* decreased firstly and then peaked at 8 h in “Yingshuang.” Expression patterns of* CsDof-10* gene rapidly declined to a low level at 2 h and then decreased gradually. High temperature stress upregulated the expression level of* CsDof-9* gradually in “Huangjinya” and downregulated in “Yingshuang.” All the expression levels of* CsDof* genes peaked at 24 h in “Huangjinya,” except for* CsDof-10* (Figure 9).

#### 3.8.2. Low Temperature (4°C) Treatment

**Figure 10:**
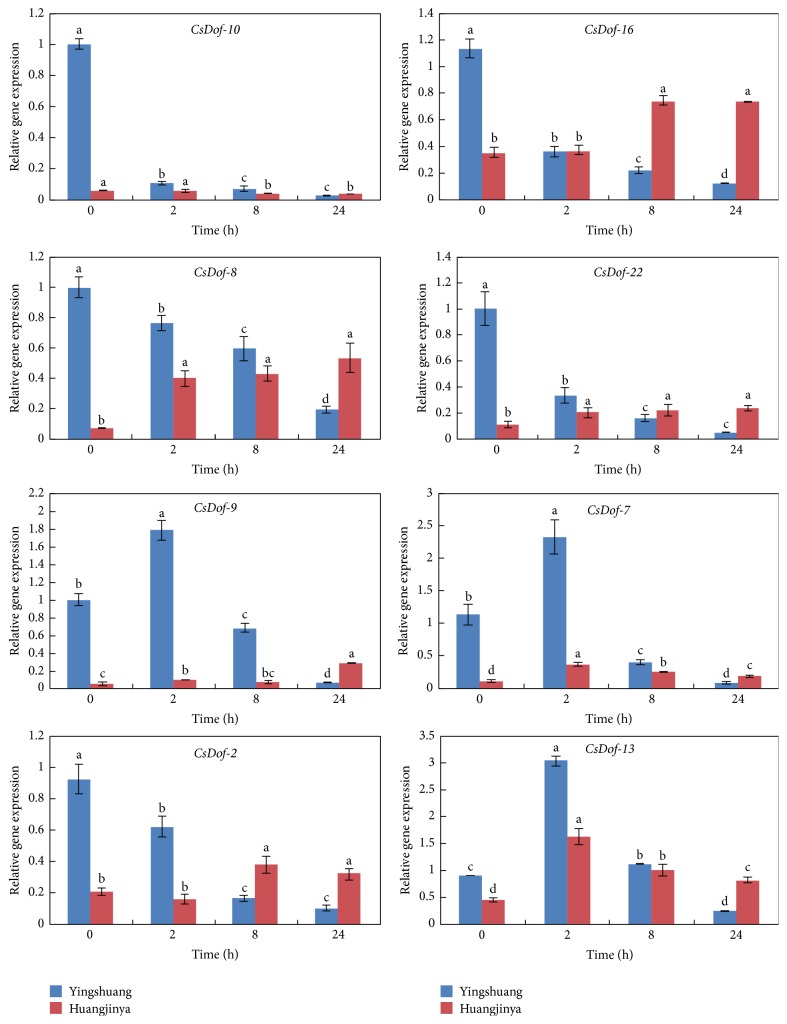
Expression patterns of* CsDof* genes in “Yingshuang” and “Huangjinya” under cold temperature stress. Error bars represent standard deviation among three real-time quantitative PCR reaction replicates. Data are means of three technical replicates ± SD. Different lowercase letters indicate significant differences at *P* < 0.05.

The* CsDof-10* was downregulated by low temperature treatment, which decreased rapidly at 2 h and then decreased gradually in “Yingshuang.” The expression level of* CsDof-10* declined to a low level gradually at 2 h, which was similar to the control in “Huangjinya.” There was a similar downward trend observed between* CsDof-8* and* CsDof-2* in “Yingshuang,” which declined firstly at 2 h and then decreased to the minimum level at 24 h. The transcript levels of* CsDof-8* and* CsDof-22* increased gradually, peaking at 5-fold and 2-fold at 24 h relative to the control in “Huangjinya,” respectively (Figure 10).

#### 3.8.3. High Salt (0.2 M NaCl) Treatment

**Figure 11:**
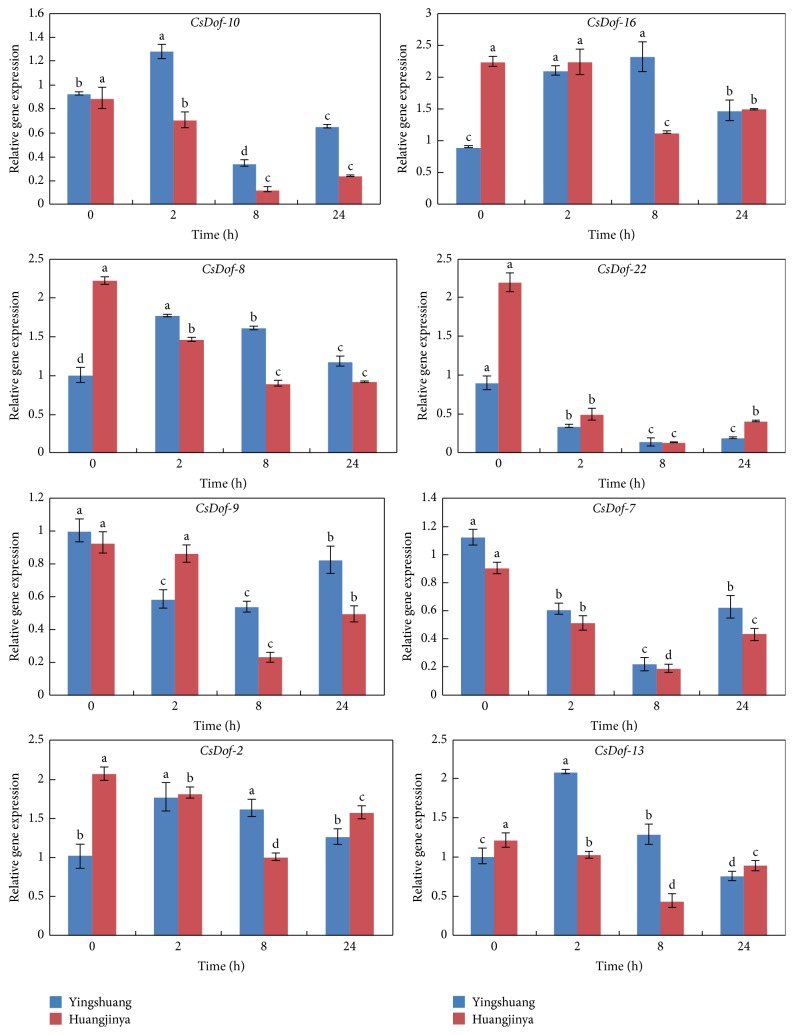
Expression patterns of* CsDof* genes in “Yingshuang” and “Huangjinya” under drought stress. Error bars represent standard deviation among three real-time quantitative PCR reaction replicates. Data are means of three technical replicates ± SD. Different lowercase letters indicate significant differences at *P* < 0.05.

The expression trend of* CsDof-7* was similar under salt treatment in “Yingshuang” and “Huangjinya,” which both decreased to the minimum level at 8 h. Meanwhile, the expression levels of* CsDof-8* and* CsDof-2* were similar in “Yingshuang” and “Huangjinya.” The transcript level of* CsDof-13* peaked at 2-fold at 2 h relative to the control and then decreased gradually in “Yingshuang” (Figure 11).

#### 3.8.4. Drought (200 g·L^−1^ PEG) Treatment

**Figure 12:**
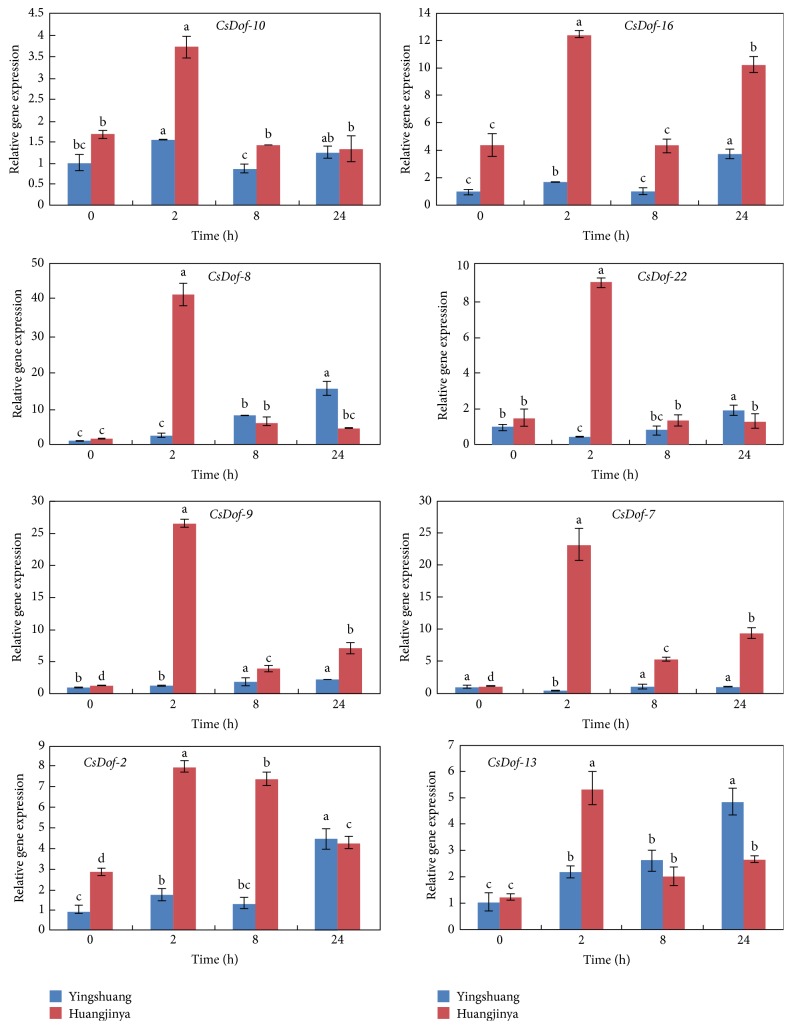
Expression patterns of* CsDof* genes in “Yingshuang” and “Huangjinya” under salt stress. Error bars represent standard deviation among three real-time quantitative PCR reaction replicates. Data are means of three technical replicates ± SD. Different lowercase letters indicate significant differences at *P* < 0.05.

All the expression levels of* CsDof* genes peaked at 2 h in “Huangjinya” under drought treatment.* CsDof-7* showed a low transcript level at 8 h, which was similar to the control in “Yingshuang.” The transcript level of* CsDof-13* increased gradually and peaked at 5-fold at 2 h relative to the control in “Huangjinya”.* CsDof-8* increased gradually and peaked at 2 h and then decreased to a low level at 24 h in “Huangjinya” (Figure 12).

## 4. Discussion

Tea plant is increasing subjected to abiotic stresses that are caused by nature during its growth and development, such as high or low temperature, salinity, and drought. These abiotic stresses have been the primary cause that leads to crop loss. The quality of tea plant has been affected by these abiotic stresses. These stresses were also accompanied by oxidative stress and might have a certain damage to the functional and structural proteins [44]. As a main source of tea beverage, tea plant should contain the excellent characteristic that has a tolerance to abiotic stresses of environment. Thus, it is intriguing and significant to analyze the TFs that are related to abiotic stresses. It might provide theoretical basis of breeding targets for tea plants.

In the present study, the tea plant transcriptome provides an important resource to analyze the regulatory roles that response to abiotic stresses in tea plant. A total of 29 CsDof TFs were identified, thereby discussed extensively by analyzing the classification as well as structure and function. The conserved domains of CsDof TFs sequences were identified, suggesting that CsDof TFs were characterized by a particular zinc finger domain. These results agreed well with the previous reports [45, 46]. Phylogenetic analysis of CsDof TFs was constructed and classified into 7 subgroups (A, B1, B2, C1, C2.1, C2.2, and D2), which directly reflected that Class D2 contained the largest number of CsDof TFs, indicating that Class D2 was one important class of Dof TFs in tea plant. The evolution of the Dof TFs in other plant species and tea plant was analyzed, and tea plants belonged to vascular plants. In the course of evolution, most of the vascular plants have developed some mechanisms to adapt to abiotic stresses [47]. In the green unicellular alga* Chlamydomonas reinhardtii*,* CrDof1* has been identified and as the first ancestral of Dof factor [48]. Although the major cause of the evolution process of* CsDof* genes in plant is unknown, some efforts have been reported that* Dof* genes multiplied during ancient days before the diversification of angiosperms [7].

The presence of conserved motifs among the* Dof* genes was investigated using MEME. There were some specific motifs in one class. For instance, motif 4, motif 5, and motif 17 were present only in Class D2, suggesting that these motifs were specific to the evolution of some members in Class D2. Recent research have showed that several motifs were confined to one Dof class [49]. Moreover, the change of conserved motifs among the new Dofs played a crucial role in the formation of the distinct subfamilies [48]. The similar motifs were included in the same class of* CsDof* genes, which might suggest that the conserved motifs and phylogenetic analysis of* CsDof* genes were mutually supported.

Dof proteins has been shown to interact with other proteins [8, 50]. The predicted protein-protein interactions between CsDof TFs and the database of* A. thaliana *were constructed. The sentence of CsDof-7 had high identity to OBP2, and other research has shown that OBP2 contains an asparagine-rich domain as well as response to auxin in* Arabidopsis* [8]. Previous research has provided evidence that OBP2 plays a role in regulating glucosinolate biosynthesis in* Arabidopsis* [16]. We found an interaction among ABA1 (ABA deficient 1), CsDof-19, and CsDof-26, which might suggest that a regulating mechanism was present among them. STO (salt tolerance protein) might act as a transcription factor in salt-stress response, and an interaction between STO and CsDof-22 was present. Thus, we might speculate that CsDof-22 play a role in salt-stress response. In addition, an interaction between ABA1 and CsDof-22 was found. The result showed the important role which ABA1 played in the ABA (abscisic acid) biosynthesis from STRING, indicating that CsDof-22 may play a similar role in ABA biosynthesis. Required for resistance to osmotic and drought stresses, there was an experimental result showing that ABA plays a role in resisting drought stress [51]. Therefore, CsDof-22 might participate in the regulation of salt-stress stimulation with different expression levels in “Yingshuang” and “Huangjinya.”

The expression profile of genes is associated with their functions. In this study, firstly, a heat map of* CsDof* genes was drawn and analyzed among four cultivars; these were produced from different areas with different environmental conditions [37]. Research has reported that nine of 36 Dof genes show a high expression level in different tissues of the* Arabidopsis* root [52]. For instance, AT3G21270 showed a higher expression level in root cap of* Arabidopsis* root.* CsDof-2* displayed a higher expression level in T1 than that in other tea plants. Moreover, we indicated that AT3G21270 and CsDof-2 belonged to Class A in the phylogenetic analysis. Regulation of* Dof* genes in response to different types of abiotic stress has been proved. For instance,* Arabidopsis* transgenic plant overexpressing* SlCDF1* or* SlCDF3* genes showed a markedly improved resistance to drought and salt [53]. Dof TFs have been reported to participate in the response to abiotic stress [21, 54]. The expression levels of eight Dof family genes were investigated under four stresses by using qRT-PCR. Under high and low temperature stresses, most* CsDof* genes showed downregulated expression levels in “Yingshuang.” However, the expression patterns were upregulated in “Huangjinya.” The results might exhibit that the expression levels of* CsDof* genes were notably different between “Yingshuang” and “Huangjinya” and were slightly influenced by extreme temperature stresses. Under salt stress, most* CsDof* genes showed downregulated expression levels in “Yingshuang” and “Huangjinya.” All the* CsDof* genes were minimally expressed at 8 h and then increased in “Huangjinya” under salt stress, which might suggest that* CsDof* genes are similarly expressed in response to salt stress in “Huangjinya.” In total, these results might improve our understanding of Dof TFs from tea plants and might play a potential role in tea plant stress resistance.

## 5. Conclusion

In conclusion, a total of 29 putative Dof TFs were identified from transcriptome of tea plant and divided into 7 groups. All of these Dof TFs contained the conserved domain. Furthermore, we found that protein-protein interactions were present among CsDof TFs and other proteins in* Arabidopsis*. After the analyzing of gene expression levels of* CsDofs* under four various abiotic stresses by qRT-PCR in “Yingshuang” and “Huangjinya,” we found that abiotic stress can cause the changes of the gene expression levels in “Yingshuang” and “Huangjinya.” Our results might suggest that there was a potential relationship between CsDof factors and tea plant stress resistance.

## Supplementary Material

Supplementary Table S1: The name and source of CsDof TFs from tea plant.
Supplementary Table S2: The coding sequences and deduced amino acid sequences of of Dof TFs from tea plant.
Supplementary Table S3: Gene codes and amino acid sequences of Dof TFs from Arabidopsis.

## Figures and Tables

**Table 1:** Primer sequences of the *Dof* genes in *C. sinensis* for qRT-PCR.

Name	Forward primer (5′–3′)	Reverse primer (5′–3′)
*CsDof-10*	GAAGCAACAGCAGCAAGATCATCAC	TTGAGCGAGTCACACCGAGGA
*CsDof-16*	ACAGGAGATAGTAGTAGTGAAACCAATGGA	CTTGGACAGTTCAAGGCTTGTTCTT
*CsDof-8*	TTGGAACAGCCAGTTTATTAGGTCTCA	TTCGCCGTAGTGATGATGATGATGAT
*CsDof-22*	GCTTCCGCTTCACATTATCGTCATATC	GAAGACCTACTTGACCGCTCATCC
*CsDof-9*	CAGAGAACATTTCGGCAAAGCAGAC	GGTGAATCACATCTTGGACACTTGAG
*CsDof-7*	TTCCACCGCCACAACAATTACCTT	ACCACCACCACCACCAGTATTAGT
*CsDof-2*	GCCGAGATACAGGAAGCAACTTAATGA	AAGCGAAACAGCCATCCAGATAGTG
*CsDof-13*	TCCATTCCTCTCATACAACTCCTTCCT	TCACTTCCACTGCCATTCATTCCATT
*Csactin*	GATTCCGTTGCCCTGAAGTCCT	CCTTGCTCATACGGTCTGCGATA

## References

[1] Arce A. L., Cabello J. V., Chan R. L. (2008). Patents on plant transcription factors. *Recent Patents on Biotechnology*.

[2] Zhang C.-Q., Xu Y., Lu Y., Yu H.-X., Gu M.-H., Liu Q.-Q. (2011). The WRKY transcription factor OsWRKY78 regulates stem elongation and seed development in rice. *Planta*.

[3] Redillas M. C. F. R., Jeong J. S., Kim Y. S. (2012). The overexpression of OsNAC9 alters the root architecture of rice plants enhancing drought resistance and grain yield under field conditions. *Plant Biotechnology Journal*.

[4] Yu L., Chen X., Wang Z. (2013). Arabidopsis enhanced drought tolerance1/HOMEODOMAIN GLABROUS11 confers drought tolerance in transgenic rice without yield penalty. *Plant Physiology*.

[5] Yanagisawa S. (2004). Dof domain proteins: plant-specific transcription factors associated with diverse phenomena unique to plants. *Plant and Cell Physiology*.

[6] Yanagisawa S., Izui K. (1993). Molecular cloning of two DNA-binding proteins of maize that are structurally different but interact with the same sequence motif. *Journal of Biological Chemistry*.

[7] Yanagisawa S. (2002). The Dof family of plant transcription factors. *Trends in Plant Science*.

[8] Kang H.-G., Singh K. B. (2000). Characterization of salicylic acid-responsive, *Arabidopsis* Dof domain proteins: overexpression of OBP3 leads to growth defects. *The Plant Journal*.

[9] Umemura Y., Ishiduka T., Yamamoto R., Esaka M. (2004). The Dof domain, a zinc finger DNA-binding domain conserved only in higher plants, truly functions as a Cys2/Cys2 Zn finger domain. *Plant Journal*.

[10] Washio K. (2003). Functional dissections between GAMYB and Dof transcription factors suggest a role for protein-protein associations in the gibberellin-mediated expression of the *RAmy1A* gene in the rice aleurone. *Plant Physiology*.

[11] Gualberti G., Papi M., Bellucci L. (2002). Mutations in the Dof zinc finger genes DAG2 and DAG1 influence with opposite effects the germination of Arabidopsis seeds. *Plant Cell*.

[12] Vicente-Carbajosa J., Moose S. P., Parsons R. L., Schmidt R. J. (1997). A maize zinc-finger protein binds the prolamin box in zein gene promoters and interacts with the basic leucine zipper transcriptional activator Opaque2. *Proceedings of the National Academy of Sciences of the United States of America*.

[13] Chen W., Chao G., Singh K. B. (1996). The promoter of a H_2_O_2_-inducible, Arabidopsis glutathione S-transferase gene contains closely linked OBF- and OBP1-binding sites. *Plant Journal*.

[14] Fornara F., Panigrahi K. C. S., Gissot L. (2009). *Arabidopsis* DOF transcription factors act redundantly to reduce CONSTANS expression and are essential for a photoperiodic flowering response. *Developmental Cell*.

[15] Imaizumi T., Schultz T. F., Harmon F. G., Ho L. A., Kay S. A. (2005). Plant science: FKF1 F-box protein mediates cyclic degradation of a repressor of CONSTANS in Arabidopsis. *Science*.

[16] Skirycz A., Reichelt M., Burow M. (2006). DOF transcription factor AtDof1.1 (OBP2) is part of a regulatory network controlling glucosinolate biosynthesis in Arabidopsis. *The Plant Journal*.

[17] Song A., Gao T., Li P. (2016). Transcriptome-wide identification and expression profiling of the DOF transcription factor gene family in *Chrysanthemum morifolium*. *Frontiers in Plant Science*.

[18] Lijavetzky D., Carbonero P., Vicente-Carbajosa J. (2003). Genome-wide comparative phylogenetic analysis of the rice and Arabidopsis Dof gene families. *BMC Evolutionary Biology*.

[19] Malviya N., Gupta S., Singh V. K. (2015). Genome wide in silico characterization of Dof gene families of pigeonpea (*Cajanus cajan* (L) Millsp.). *Molecular Biology Reports*.

[20] Shu Y. J., Song L. L., Zhang J., Liu Y., Guo C. H. (2015). Genome-wide identification and characterization of the dof gene family in *Medicago truncatula*. *Genetics and Molecular Research*.

[21] Ma J., Li M.-Y., Wang F., Tang J., Xiong A.-S. (2015). Genome-wide analysis of Dof family transcription factors and their responses to abiotic stresses in Chinese cabbage. *BMC Genomics*.

[22] Guo Y., Qiu L.-J. (2013). Genome-wide analysis of the dof transcription factor gene family reveals soybean-specific duplicable and functional characteristics. *PLoS ONE*.

[23] Wei P.-C., Tan F., Gao X.-Q. (2010). Overexpression of AtDOF4.7, an arabidopsis DOF family transcription factor, induces floral organ abscission deficiency in arabidopsis. *Plant Physiology*.

[24] Sugiyama T., Ishida T., Tabei N. (2012). Involvement of PpDof1 transcriptional repressor in the nutrient condition-dependent growth control of protonemal filaments in Physcomitrella patens. *Journal of Experimental Botany*.

[25] Xu J., Dai H. (2016). *Brassica napus* Cycling Dof Factor1 (*BnCDF1*) is involved in flowering time and freezing tolerance. *Plant Growth Regulation*.

[26] Rueda-López M., Cañas R. A., Canales J., Cánovas F. M., Ávila C. (2015). The overexpression of the pine transcription factor PpDof5 in Arabidopsis leads to increased lignin content and affects carbon and nitrogen metabolism. *Physiologia Plantarum*.

[27] Wu Q., Li D., Li D. (2015). Overexpression of *OsDof12* affects plant architecture in rice (*Oryza sativa* L.). *Frontiers in Plant Science*.

[28] Wu Z., Cheng J., Cui J. (2016). Genome-wide identification and expression profile of dof transcription factor gene family in pepper (*Capsicum annuum* L.). *Frontiers in Plant Science*.

[29] Yamamoto T., Juneja L., Chu D. C. (1997). *Chemistry and Applications of Green Tea*.

[30] Chen Y., Yu M., Xu J., Chen X., Shi J. (2009). Differentiation of eight tea (*Camellia sinensis*) cultivars in China by elemental fingerprint of their leaves. *Journal of the Science of Food and Agriculture*.

[31] Bordoni A., Hrelia S., Angeloni C. (2002). Green tea protection of hypoxia/reoxygenation injury in cultured cardiac cells. *Journal of Nutritional Biochemistry*.

[32] Levites Y., Youdim M. B. H., Maor G., Mandel S. (2002). Attenuation of 6-hydroxydopamine (6-OHDA)-induced nuclear factor-kappaB (NF-*κ*B) activation and cell death by tea extracts in neuronal cultures. *Biochemical Pharmacology*.

[33] Nie G., Jin C., Cao Y., Shen S., Zhao B. (2002). Distinct effects of tea catechins on 6-hydroxydopamine-induced apoptosis in PC12 cells. *Archives of Biochemistry and Biophysics*.

[34] Wu Z.-J., Li X.-H., Liu Z.-W., Li H., Wang Y.-X., Zhuang J. (2015). Transcriptome-based discovery of AP2/ERF transcription factors related to temperature stress in tea plant (*Camellia sinensis*). *Functional and Integrative Genomics*.

[35] Liu Z.-W., Wu Z.-J., Li X.-H. (2016). Identification, classification, and expression profiles of heat shock transcription factors in tea plant (*Camellia sinensis*) under temperature stress. *Gene*.

[36] Riaño-Pachón D. M., Ruzicic S., Dreyer I., Mueller-Roeber B. (2007). PlnTFDB: an integrative plant transcription factor database. *BMC Bioinformatics*.

[37] Wu Z.-J., Li X.-H., Liu Z.-W., Xu Z.-S., Zhuang J. (2014). De novo assembly and transcriptome characterization: novel insights into catechins biosynthesis in *Camellia sinensis*. *BMC Plant Biology*.

[38] Bailey T. L., Boden M., Buske F. A. (2009). MEME Suite: tools for motif discovery and searching. *Nucleic Acids Research*.

[39] Szklarczyk D., Franceschini A., Wyder S. (2015). STRING v10: protein-protein interaction networks, integrated over the tree of life. *Nucleic Acids Research*.

[40] Deng W. K., Wang Y. B., Liu Z. X., Cheng H., Xue Y. (2014). HemI: a toolkit for illustrating heatmaps. *PLoS ONE*.

[41] Chenna R., Sugawara H., Koike T. (2003). Multiple sequence alignment with the Clustal series of programs. *Nucleic Acids Research*.

[42] Tamura K., Peterson D., Peterson N., Stecher G., Nei M., Kumar S. (2011). MEGA5: molecular evolutionary genetics analysis using maximum likelihood, evolutionary distance, and maximum parsimony methods. *Molecular Biology and Evolution*.

[43] Pfaffl M. W. (2001). A new mathematical model for relative quantification in real-time RT-PCR. *Nucleic acids research*.

[44] Smirnoff N. (1998). Plant resistance to environmental stress. *Current Opinion in Biotechnology*.

[45] Yanagisawa S. (1996). A novel multigene family that the gene for a maize DNA-binding protein, MNB1a belongs to: isolation of genomic clones from this family and some aspects of its molecular evolution. *Biochemistry and Molecular Biology International*.

[46] Yanagisawa S., Schmidt R. J. (1999). Diversity and similarity among recognition sequences of Dof transcription factors. *The Plant Journal*.

[47] Wang X., Liu Y., Yang P. (2012). Proteomic studies of the abiotic stresses response in model moss—*Physcomitrella patens*. *Frontiers in Plant Science*.

[48] Moreno-Risueno M. Á., Martínez M., Vicente-Carbajosa J., Carbonero P. (2007). The family of DOF transcription factors: from green unicellular algae to vascular plants. *Molecular Genetics and Genomics*.

[49] Noguero M., Atif R. M., Ochatt S., Thompson R. D. (2013). The role of the DNA-binding One Zinc Finger (DOF) transcription factor family in plants. *Plant Science*.

[50] Zhang B., Chen W., Foley R. M. (1995). Interactions between distinct types of DNA binding proteins enhance binding to ocs element promoter sequences. *Plant Cell*.

[51] Zhou L., Xu H., Mischke S. (2014). Exogenous abscisic acid significantly affects proteome in tea plant (*Camellia sinensis*) exposed to drought stress. *Horticulture Research*.

[52] Gardiner J., Sherr I., Scarpella E. (2010). Expression of DOF genes identifies early stages of vascular development in *Arabidopsis* leaves. *International Journal of Developmental Biology*.

[53] Corrales A.-R., Nebauer S. G., Carrillo L. (2014). Characterization of tomato Cycling Dof Factors reveals conserved and new functions in the control of flowering time and abiotic stress responses. *Journal of Experimental Botany*.

[54] Huang W., Huang Y., Li M.-Y., Wang F., Xu Z.-S., Xiong A.-S. (2016). Dof transcription factors in carrot: genome-wide analysis and their response to abiotic stress. *Biotechnology Letters*.

