# Supplementary material for: Transcriptome-Based Analysis of Dof Family Transcription Factors and Their Responses to Abiotic Stress in Tea Plant (Camellia sinensis)
Source: Int J Genomics. 2016 Oct 31;2016:5614142. doi: 10.1155/2016/5614142 (PMC5107859; doi:10.1155/2016/5614142)
Supplement: Supplementary file 1 — Supplementary Table S1: The name and source of CsDof TFs from tea plant. Supplementary Table S2: The coding sequences and deduced amino acid sequences of of Dof TFs from tea plant. Supplementary Table S3: Gene codes and amino acid sequences of Dof TFs from Arabidopsis. [file 5614142.f1.doc]

**Supplementary Tables**

**Table S1 The name and source of CsDof TFs from tea plant**

| No. | Subfamily | name |
| --- | --- | --- |
| 1 | CsDof-A | *CsDof-1* |
| 2 |  | *CsDof-2* |
| 3 |  | *CsDof-3* |
| 4 |  | *CsDof-4* |
| 5 | CsDof-B1 | *CsDof-5* |
| 6 |  | *CsDof-6* |
| 7 |  | *CsDof-7* |
| 8 |  | *CsDof-8* |
| 9 |  | *CsDof-9* |
| 10 | CsDof-B2 | *CsDof-10* |
| 11 |  | *CsDof-11* |
| 12 | CsDof-C1 | *CsDof-12* |
| 13 |  | *CsDof-13* |
| 14 | CsDof-C2.1 | *CsDof-14* |
| 15 |  | *CsDof-15* |
| 16 |  | *CsDof-16* |
| 17 |  | *CsDof-17* |
| 18 | CsDof-C2.2 | *CsDof-18* |
| 19 | CsDof-D2 | *CsDof-19* |
| 20 |  | *CsDof-20* |
| 21 |  | *CsDof-21* |
| 22 |  | *CsDof-22* |
| 23 |  | *CsDof-23* |
| 24 |  | *CsDof-24* |
| 25 |  | *CsDof-25* |
| 26 |  | *CsDof-26* |
| 27 |  | *CsDof-27* |
| 28 |  | *CsDof-28* |
| 29 |  | *CsDof-29* |

**Table S2** The coding sequences and deduced amino acid sequences of of Dof TFs from tea plant

| Gene | Coding sequences | Deduced amino acid sequences |
| --- | --- | --- |
| *CsDof-1* | ATGCAAGATATACATTCGATTGGAGGCGGCGGCGGCGGAGACAGGCGTATGAGGCCGCACCACCACCAGAACCACCAGTCCTTGAAGTGTCCCCGGTGCGATTCTCTCAACACCAAGTTCTGCTACTACAACAACTACAACCTCTCTCAGCCCCGCCACTTCTGCAAGAACTGCCGCCGGTACTGGACCAAAGGCGGCGTCCTCCGCAACGTTCCCGTCGGCGGCGGCTGCCGGAAAACCAAACGCTCTAAGCCTAAAACCTCCTCCGCCACCGCCACCGCCGCCGCCACCGCCGATGCGCCTCGAGATCGCAAATCGAACTCTCATTCCAGCAGCGAGAGCTCCAGCCTCACCGCCTCAACCACCGCCGCGCCGCCGTCCACCGTCGAGGTAGCGTCGGCGAGCTCTTCGACATCGGCGTCGACATTACTCAACTTTCCGGACTCGAGGTTCTTCAATAGTATTCCACAAGCTACAAACCCTAGCTTCCGTCATCATCATCATCCGCCGTTGATCGATCAGTCATCATCGGACGGCGGGATCTTCCCGGAGATCGGAGGTAGTTTCACCAGCCTGATGACGTCATCCAACGATTCATCGTCGATTCTAGGGTTCAACATCGCTGACATTTCGGCTTCGGCGTACCGGTTGCACCAACAGAATCAGGGACCGGTTGTGCAGAATCAGAATTCGAATCAGATATGGCAGGAACAGCAGGAGAAGATGGGGTGTTCGGTACCTAATGAATTGAAGATGCAGCAGATCTCGGCCGGGTTTCTGGATCAGACTGCTGAGGTTGATTTATCGGGTATGCAGAACAGAGAGAACAGCGGTGGGCTAGGAGGACTTGATTGGCAAGTTGGTGGTGATCAAGGGTTGTTTGATTTTACTGGAACCGTTGATCAAGCTTACTGGAGTCAAACTCAGTGGAATGACAGCGATCACCATCTTTATCTCCCA | MQDIHSIGGGGGGDRRMRPHHHQNHQSLKCPRCDSLNTKFCYYNNYNLSQPRHFCKNCRRYWTKGGVLRNVPVGGGCRKTKRSKPKTSSATATAAATADAPRDRKSNSHSSSESSSLTASTTAAPPSTVEVASASSSTSASTLLNFPDSRFFNSIPQATNPSFRHHHHPPLIDQSSSDGGIFPEIGGSFTSLMTSSNDSSSILGFNIADISASAYRLHQQNQGPVVQNQNSNQIWQEQQEKMGCSVPNELKMQQISAGFLDQTAEVDLSGMQNRENSGGLGGLDWQVGGDQGLFDFTGTVDQAYWSQTQWNDSDHHLYLP |
| *CsDof-2* | ATGCAAGATATACATTCGATTGGAGGCGGCGGCGGCGGAGACAGGCGTATGAGGCCGCACCACCACCAGAACCACCAGTCCTTGAAGTGTCCCCGGTGCGATTCTCTCAACACCAAGTTCTGCTACTACAACAACTACAACCTCTCTCAGCCCCGCCACTTCTGCAAGAACTGCCGCCGGTACTGGACCAAAGGCGGCGTCCTCCGCAACGTTCCCGTCGGCGGCGGCTGCCGGAAAACCAAACGCTCTAAGCCTAAAACCTCTTCCGCCACCGCCACCGCCGCCGCCACCGCCACCGCTCTCCCTTCGTACAGGCCTTTAGGCGCAGCGACTTCGATGGTTTCGCGAGGCGCATGGCCACCGGCGAGGTCTGGAGGGACGTGTGGCGGAGCGCCAACGACGGCTTCGAGCAACTCCTTTATGAGACCAAGAAGACGGCGGAGCGCATCGACCGTCGGTACTCCGTCTCGCGGCGGCTTTCGGCGGTCGCCCAATCCGCCTCCTACCGGGCCAGAGAGATCGACCGCGACTTGGAGATCACGCAGCGTTGGCGGACTTTTACCCTCGATTTCAGTCGGAATTGGCCGAGATACAGGAAGCAACTTAATGACTTTTCAAACACTCCTATTGGAAAAAGTTTTGCTACAATATTCTTCATCTGGTTTGCACTATCTGGATGGCTGTTTCGCTTCT | MQDIHSIGGGGGGDRRMRPHHHQNHQSLKCPRCDSLNTKFCYYNNYNLSQPRHFCKNCRRYWTKGGVLRNVPVGGGCRKTKRSKPKTSSATATAAATATALPSYRPLGAATSMVSRGAWPPARSGGTCGGAPTTASSNSFMRPRRRRSASTVGTPSRGGFRRSPNPPPTGPERSTATWRSRSVGGLLPSISVGIGRDTGSNLMTFQTLLLEKVLLQYSSSGLHYLDGCFAS |
| *CsDof-3* | ATGCAAGACCCATCAATGTATCAGCAAATGAATCCCCAATTCCCAGAACACGAGCAGCTAAAATGTCCACGGTGTGAATCAACGAACACCAAATTCTGTTACTACAACAACTACAATACGTCACAACCACGCCACTTTTGCCGGAACTGCAAGAGGTACTGGACTAAAGGAGGCACTCTTCGCAACGTACCAGTCGGCGGTGGCACTCGCAGCAAGAACACCAAGCACACTTCTTCAAACCCCAAACGCTCTTCTTCTTTTTCTTCT | MQDPSMYQQMNPQFPEHEQLKCPRCESTNTKFCYYNNYNTSQPRHFCRNCKRYWTKGGTLRNVPVGGGTRSKNTKHTSSNPKRSSSFSS |
| *CsDof-4* | ATGCAAGACCCATCAACGTATCAACAGATCCAACCCCAATTCCCCGAACACGAGCACTTGAAATGCCCAAGATGTGATTCAACAAACACCAAATTTTGCTACTACAACAACTACAATCTCTCTCAGCCTCGCCACTTCTGCAAGAACTGCCGGAGATACTGGACCAAAGGCGGAGCTCTTCGCAACATCCCAATCGGCGGTGGGAGTCGCAAGAACACGAAGCGAAATTCGAACCCTAAACGCTCTTCATCGTCGTCTTCTTCGTCCTCCACCACCACAGCAACAACCACCCAAGTCGCCGGAAAACCAGAACCCTCCTCTGCCATGTACGGTTTCGCTGCGGCGGCGTTCGATCAGGAGCAGCATCGGATGATGGATATCAATGGGAGTTTTAGCTCGCTTCTGGCTTCGAGTGGGCAGTTTGGGAATTTGATGGAGGGTTTGAATCCAAATGGATCGACGGTTCGATTGGGGGAGTTTGGCGAGAATAATTTGAATTTGAATTCGAATTTGGAGGCGAATTTGATGAGAAACTCACAAGTTGAGTTACAGAGTAGTAATAATTCAGAGAGCTTTTTGGGTGTTCAGAGTGGTGGTGGTGGTGGTGGTGATTGGGGTGGTGGCAATGGGTGGCCCGATCTTGCTATTTACACACCAGGTTCGACTTTTCAG | MQDPSTYQQIQPQFPEHEHLKCPRCDSTNTKFCYYNNYNLSQPRHFCKNCRRYWTKGGALRNIPIGGGSRKNTKRNSNPKRSSSSSSSSSTTTATTTQVAGKPEPSSAMYGFAAAAFDQEQHRMMDINGSFSSLLASSGQFGNLMEGLNPNGSTVRLGEFGENNLNLNSNLEANLMRNSQVELQSSNNSESFLGVQSGGGGGGDWGGGNGWPDLAIYTPGSTFQ |
| *CsDof-5* | ATGGTTTTCTCATCTGTTCCAGTCTACTTGGATCATCCCAATTGGCACCACCAGTTACAGCAACAAAATTACCATCATCAACAAGGAGGTGTCACTGAAAATTCTCAACTTCAGCCCCCACCCCCATCCCCACCACCTCAACTGAGTGGTGGTATTGGCGGCAGCGATGGTGGCTCAGTCAGACCAGGTTCCATGGTCGATCGAGCTCGGATAGCAAAGATACCGCTTCCAGAGGCAGGTCTAAACTGCCCGCGATGCGATTCCACTAATACTAAATTTTGCTACTTTAACAATTACAGCCTCTCTCAGCCGCGACACTTCTGCAAGACGTGTCGCCGGTACTGGACAAGAGGCGGTGCCCTAAGGAGTGTTCCGGTGGGTGGTGGCTGCCGGAGAAGCAAGAAAATCAAGAACAACAGTAGCTCAAAATCTCAGGTCACATCTGAGAACCGAACAAGGCCTAATTCTAAGTCAACAAGTGCCAGTCCCTCTAGCTGCAGCACAGATAATAATATATCTGGTGGTTCTGGTCATTTTCCTCCACCACCACAATTTCCATTCATGGCAGCTCTACAAAACCTAGCTCAGTACGGTGGTGGTGGTGGAGGGAACAATATTGGGTCAAATATCGCTGAATTTCAGGCGCAGATGGCGGCAGCAGGTGGTGGTGGAGGGGTGGAGCAGTGGAGGTTGCCTTTCTTGGGTGGTCTTGAGACACCCACAAATTTGTTGTACCCGTCCTACCAAAGTCAAGGATTTGAAGCACAATCTTCTGGTATTAGTACTCAGATTGGTTCAGTGAAAATGGAAGAGAATATGAACAACCAAGGGCTTAATTTTCCAAGGCAGTTTATGGGTATGACAGAAAATTACCAGTACTGTGGTGGGATTGGGAATACATGGAATGAGTTTGCTGGTGTAAACTCATCTTCCACCAGTCATCTCTTA | MVFSSVPVYLDHPNWHHQLQQQNYHHQQGGVTENSQLQPPPPSPPPQLSGGIGGSDGGSVRPGSMVDRARIAKIPLPEAGLNCPRCDSTNTKFCYFNNYSLSQPRHFCKTCRRYWTRGGALRSVPVGGGCRRSKKIKNNSSSKSQVTSENRTRPNSKSTSASPSSCSTDNNISGGSGHFPPPPQFPFMAALQNLAQYGGGGGGNNIGSNIAEFQAQMAAAGGGGGVEQWRLPFLGGLETPTNLLYPSYQSQGFEAQSSGISTQIGSVKMEENMNNQGLNFPRQFMGMTENYQYCGGIGNTWNEFAGVNSSSTSHLL |
| *CsDof-6* | ATGGTTTTCTCATCTGTTCCACTCTATTTAGATCAACCCAATTGGCACCAGTTGCAGCAACCAAACCATCAAGAAGTATGTGGTGGCGAAAATTCTCAGTTTCAGCCGCCGCCGCCACCTCAAGTGGGTGTTGGTTGCGGTGGTGGTGGCTCAATCAGACCTGGTTCGATGGTTGATCGAGCACGGATAGCGAAGATTCCTCAGCCAGAGGCAGGACTTAAGTGCCCGCGATGCGAATCAACCAATACAAAATTTTGCTACTTCAATAACTACAATCTCACGCAGCCCCGGCACTTCTGCAAGACGTGTCGCCGGTACTGGACACGAGGTGGTGCTCTAAGGAATGTTCCGGTGGGTGGAGGCTGCCGGAGAAACAAGAAAATCAAGAACAGTAGTAGCTCAAAATCTCAGGTCACCGCCGATCAAAAGGCAGTGGTAGGGCTTAAATCCACAAGCCCAAGTCCAAGCCCCTCTAGCTGCAACAACAACACAGACATGCCTAGTAGTCATTTTACACAATTACCCTTCATGGATTCGGCTTTACAAAACCTAGGTCAGTATGGCGGTGGAGGGGGTAATTTGGGTATTATTAGTGGTGGATTTCAGGCACAAATGGCGGCTACTGGTGGTGCTGGGCTGACGAGTTTAGGGTTTCAGATATCGGGTGGTGGAGGAGGAGCTGATCAGTGGCGATTGCCTTTCTTGACCGGTCTTGAAACTCCGGGGATTTTTTACCCGCATCATCAAAGTGAAGTTGTTCAAAATCGATCTTCGGGACAGGTTGAGACGGTGGCACCACTGGGTGGTGGTGGTTCTGGTGTTGCTCTGGTGGCTGCTCCGGTGCAAATGGAAGAGAAAAACCGAGGAGGGCTTAACTTGTCAAGACAGTTTATGGGTATTTTTGAGAATAATAATAATAATAGTCAGTACTTGGGTGGGAATACTTGGACTGAAATTTCTGGAATGAATTCTTCT | MVFSSVPLYLDQPNWHQLQQPNHQEVCGGENSQFQPPPPPQVGVGCGGGGSIRPGSMVDRARIAKIPQPEAGLKCPRCESTNTKFCYFNNYNLTQPRHFCKTCRRYWTRGGALRNVPVGGGCRRNKKIKNSSSSKSQVTADQKAVVGLKSTSPSPSPSSCNNNTDMPSSHFTQLPFMDSALQNLGQYGGGGGNLGIISGGFQAQMAATGGAGLTSLGFQISGGGGGADQWRLPFLTGLETPGIFYPHHQSEVVQNRSSGQVETVAPLGGGGSGVALVAAPVQMEEKNRGGLNLSRQFMGIFENNNNNSQYLGGNTWTEISGMNSS |
| *CsDof-7* | ATGGTTTTCTCATCCGTTCCAGTCTATCTAGATCCTCCTCATCACAACTGGCAACAACAACAGCTTCCAAATCACCACCAATCCGGAGGTGGCGAGAGTGATAATAATCACTTTCCACCGCCTCTTCCGCCACCTCCAAGTGGTGGAAGTGGTGGCACCGCCACGGGCTCAACCAGGCCAGGTTCGATGGCGGAGCGGGCCAGGCTAGCCAAGATGCCACAGCCTGAGGTAGCACTTAAGTGCCCTAGATGTGAATCAACCAACACAAAGTTTTGCTATTTCAATAACTATAGTCTCACACAGCCACGTCACTTCTGCAAGACTTGCCGGCGGTACTGGACTCGAGGCGGTGCACTGAGAAGCGTGCCGGTCGGAGGTGGCTGCCGGAGAAACAAGAAAAGCAAAAGAAACAGATCAAAATCACCGGCAATGAATGATCACCGCCGTGATGGTTCTAGTTCTGCTGCCGGAGTTACGTCCACCACCACCTGCACCACCGGTATGATTGGCCACTTGATCCTTCCACCGCCACAACAATTACCTTTTTTGCCCTCTTTGCCTCACCTTAGCGACTACAGCAGCACCGATATTTCATTGAATTTCCCGGGAATTCAGCCTCCAGTGGCGGCAACAGGCGGTGTTAATGGCGGAATCGATATGGAATTCGGAATCGGAACTAATACTGGTGGTGGTGGTGGT | MVFSSVPVYLDPPHHNWQQQQLPNHHQSGGGESDNNHFPPPLPPPPSGGSGGTATGSTRPGSMAERARLAKMPQPEVALKCPRCESTNTKFCYFNNYSLTQPRHFCKTCRRYWTRGGALRSVPVGGGCRRNKKSKRNRSKSPAMNDHRRDGSSSAAGVTSTTTCTTGMIGHLILPPPQQLPFLPSLPHLSDYSSTDISLNFPGIQPPVAATGGVNGGIDMEFGIGTNTGGGGG |
| *CsDof-8* | ATGGCCAACATACCCATGCCAGAAGCTGCCCTAAAATGCCCGAGATGTGAATCAACAAACACAAAGTTTTGCTACTTCAACAACTACAGCCTCACTCAGCCTCGGCACTTTTGCAAGACTTGCCGGAGGTACTGGACGAGAGGCGGCGCTCTGAGAAGCGTTCCAGTGGGCGGTGGTTGCCGGAGAAACAAGAGAAGCAAAGGAAGTAACAGCTCGAAATCTCCGGTCAGCGGTGGCGGTGGTGATGGGCAAACTAGCAGTGGTTCAACGAGTGCAATCTCATCTTCTACTAGTGTTGGAACAGCCAGTTTATTAGGTCTCACACCACAAATTCCACCACTCCGGTTCATGGCAGCTCCACTGAGTCAACTCACTGATCATCATCATCATCACTACGGCGAAATCGGGTTGAATTACAGTGGAATTTCAGCTCCTTTGGTGGTTACAAGTGACATGAACATGAATTTCCACCTTGGAAGTAGTTCACTTGGTCTCGGTGGCGGTGGCAGTGGAATCGAGCAGTGGCGGTTGCAACAACCAACTCAGATTTCCCCAATGGCGGGCTTGGATTTTCCGGGTGTTTTGTACCCATTTCAAGGTGGTGTAGAGCCCTCTGGCTATGGTGGTGAGGGTAATCAGGTCCGGCCAAAGCCGCCCGGTTTAGGGTTTACTACTCATCAACTCGGTTCAGTGAAAATGGAAGACAATCAGGTTCAAGAGTTGAATTTGTCAAGGCAGTTTTTGGGAATTCCAGGAAATGATCAGTATTGGAGTGGTTCTAGTTCTGCTTGGACTGACCTTTCTGGGTTTAGCTCATCTTCCACTAGGAATCCCCTA | MANIPMPEAALKCPRCESTNTKFCYFNNYSLTQPRHFCKTCRRYWTRGGALRSVPVGGGCRRNKRSKGSNSSKSPVSGGGGDGQTSSGSTSAISSSTSVGTASLLGLTPQIPPLRFMAAPLSQLTDHHHHHYGEIGLNYSGISAPLVVTSDMNMNFHLGSSSLGLGGGGSGIEQWRLQQPTQISPMAGLDFPGVLYPFQGGVEPSGYGGEGNQVRPKPPGLGFTTHQLGSVKMEDNQVQELNLSRQFLGIPGNDQYWSGSSSAWTDLSGFSSSSTRNPL |
| *CsDof-9* | ATGATGTCCGCAGAGAACATTTCGGCAAAGCAGACCTCAAAAGATGAAAGCCAGAGCTCCAGCAGCCGTAAAACCACATCGATGAGGCCACCAGAACAAGCCCTCAAGTGTCCAAGATGTGATTCACCCAACACAAAGTTCTGCTACTACAAC | MMSAENISAKQTSKDESQSSSSRKTTSMRPPEQALKCPRCDSPNTKFCYYN |
| *CsDof-10* | ATGGAGCAAGAAGAGGGTATGAAGCAACAGCAGCAAGATCATCACCACCACCACCACCGGCGGCTGAAGCCACCGCAGACGGCGGTGCAATCGGACCAACAGACACCGCAGCCGCAGCAGCAGCCGCAGAAGTGTCCTCGGTGTGACTCGCTCAACACCAAGTTCTGCTACTAC | MEQEEGMKQQQQDHHHHHHRRLKPPQTAVQSDQQTPQPQQQPQKCPRCDSLNTKFCYY |
| *CsDof-11* | ATGGAGAAACCAATCCAAGACCAAAGCCAAATCCAAATTCATCAAGCCCTCAGGTGTCCTCGCTGTGATTCATCAAACACCAAGTTCTGTTACTACAAC | MEKPIQDQSQIQIHQALRCPRCDSSNTKFCYYN |
| *CsDof-12* | ATGGATCCTTCTAGTGCACAACACCATCAGGAGATCGCAACTCATCAAACCCTAGAAAGCATGTTGGTTTGCACAAAAACAAATCAAGAGAAGAAACCAAGGCCTCAGCCAGAACAAGCACAGAAGTGCCCTAGATGTGAATCCACCAACACCAAGTTTTGTTACTACAACAACTACAGTCTCTCTCAGCCAAGGTACTTTTGCAAGTCATGCAAGAGGTATTGGACCAAAGGAGGAACACTGAGAAATGTTCCAGTAGGTGGAGGATGCAGGAAGAACAGGAGGTCCTCACCACCATCAAAGAGGAGCCAAGATGATCATCAACCACTGATCCCAACCCATCACAATCCCCTCTCTACTCTCCCTTGCTTGACCTATGATTCCAATGATCTCAGCCTTGCATTTGCTAGGCTCCACAATCAACCAAATGGTCATCTAGGGTTTGATCATGATCATCATCATCATGATCAGGGTGTTCCAATGTTTGATGTTCTTGGAAACCCTAATGTCCAAACCTATGGTACAACTCCTCATGCCTTTCTTGAAGCCCCAAATGGCTTTCATGGTTTGTACTATGGGATGATGGGTAATGGGGGTGGTGCGAGTGTCAATGTTAGTGGAGAAATGGTGAGGCCATATGAAGATATGACAGTGACAACAATGAAGCAAGAGTTGAGCAATGGAAGAGAAGGAGAGAATAGAGTGTTGTGGGGATTCCCATGGCAAATTAGTGGAGATGCAAACATGGGTGATGTTGATTCAGGGAGAGAGAGTTGGAATGCATTTGGTTCATCTTGGCATGGACTTCTCCATAGCCCTCTAATG | MDPSSAQHHQEIATHQTLESMLVCTKTNQEKKPRPQPEQAQKCPRCESTNTKFCYYNNYSLSQPRYFCKSCKRYWTKGGTLRNVPVGGGCRKNRRSSPPSKRSQDDHQPLIPTHHNPLSTLPCLTYDSNDLSLAFARLHNQPNGHLGFDHDHHHHDQGVPMFDVLGNPNVQTYGTTPHAFLEAPNGFHGLYYGMMGNGGGASVNVSGEMVRPYEDMTVTTMKQELSNGREGENRVLWGFPWQISGDANMGDVDSGRESWNAFGSSWHGLLHSPLM |
| *CsDof-13* | ATGGATCCTTCAAGTGCACAACACCACCAGGAAATGGGTACTCAAACCTTGGAAAGTATGTTGGGTTGCACAAAAGCACAGCAAGAGAAGAAACCAAGGCCTCAGCCAGAACAAGCTCTGAAATGCCCTAGATGTGACTCTACCAACACCAAGTTTTGTTACTACAACAACTACAGCCTCACTCAGCCAAGGTACTTCTGCAAGTCATGCAGGAGGTACTGGACCAAAGGGGGAACTTTGAGAAATGTTCCAGTGGGTGGAGGCTGCAGGAAGAACAAGAGATCATCATCATCATCATCATCAAAGAGGAGCCAAGATCAAGGCCTCACTACCAATCCCAGCCCTCTATCAAATCTCCCAACCTTGGGCTATGATTCTAATGATCTCAGCCTTGCATTTGCTAGGCTGCAAAAGCACCCAACTGGGCAGCTAGGGTTTGATGAGCATGATTTTTCAATGTTGGGAAGCCATGGAAATACACATTGTGATGTTCTTGGAAACCCTAATGTCCATTCCTCTCATACAACTCCTTCCTTTCTTGATGCACTTAGGAGTGGATTTCTTGAAGCCCCAAATGGGTTTCACAATATGTATTATGGGATGAGTAATGGAAACATGGGTCAAGTGGAAAATGGAATGAATGGCAGTGGAAGTGAAGAAATGGGAATTCCATATGAAGATGTGAGTGGTGGTGCAACAACAACAGCGGTAACGGTGACGACAATGAAGCAAGAGTTGTGCAATGGGAGAGAGGGAGAGAATAGGGTTTTGTGGGGATTTCCATGGCAGATTGGTGGAGATGGAAACATGGTGGGTGATCTTGTTGATTCAGGAAGAGAAAGCTGGAATGGACTTGGTGTTGGTCTTGGTGTTGGTGGTGGTTCATCTTGGCATGGACTTCTCAATAGCCCTCTCATG | MDPSSAQHHQEMGTQTLESMLGCTKAQQEKKPRPQPEQALKCPRCDSTNTKFCYYNNYSLTQPRYFCKSCRRYWTKGGTLRNVPVGGGCRKNKRSSSSSSSKRSQDQGLTTNPSPLSNLPTLGYDSNDLSLAFARLQKHPTGQLGFDEHDFSMLGSHGNTHCDVLGNPNVHSSHTTPSFLDALRSGFLEAPNGFHNMYYGMSNGNMGQVENGMNGSGSEEMGIPYEDVSGGATTTAVTVTTMKQELCNGREGENRVLWGFPWQIGGDGNMVGDLVDSGRESWNGLGVGLGVGGGSSWHGLLNSPLM |
| *CsDof-14* | ATGGATACTGCTCAATGGCCTCAGGGTTTTCAGGAGGTTCGGATGGTTGATCCCAAGGAAGAGACGCCTATGATGTTGGAGAGGAAGGTAAGGCCACAAAAGGATCAAGCCTTGAATTGTCCAAGGTGCAATTCAACCAAC | MDTAQWPQGFQEVRMVDPKEETPMMLERKVRPQKDQALNCPRCNSTN |
| *CsDof-15* | ATGGATACTAGTGCTCAGTGGCCACAGGGGGTAGGGTTGGTGAAACCCATGGAAGGCTCAAGGGCTATAACTGAAAGGAAGCCAAGACCACAAAAGGAACAAGCTTTGAACTGCCCTAGGTGCAATTCAACCAACACCAAGTTTTGTTACTACAATAATTACAGTCTCACTCAACCAAGGTACTTTTGTAAGACTTGTAGAAGATATTGGACTGAAGGAGGGTCTCTCAGAAACGTTCCAGTTGGAGGAGGTTCAAGGAAGAACAAGAGATCATCGTCGTCGTCATCACCATCACCATCATCAAAAAAGCTTCCCGATCATCTGGGCCCACCAAGTTTCTCATCTCAGTCTGCCTCTCAAAACCCTAGGATCCATGAAAACCTCAACCTAGCTTACCCAGCAAGTACAAGTGATAATTACCATAATGCCCTTAATCCTAACCCTTCCTCTACTTCCCCTCATGTGTCAGCCATGGAGCTTCTCAAGACTGGGATTTCTTCAAGGGGACTCAGTTCTTTCATGCCGATGCCAATTCCGGACTCGAATTCAATCTACCCATCTGGGTTTCCTATGCAAGAATTCAAGCCAAGCCTCAATTTTTCTCTAGATGGGTTTGAAAGTGGGTATCATCATCATCATCATCAAGAAAATAGTAGCGCGAGGTTGTTTTTTCCATTTGAGGAATTGAAGCAAGCACCAAACACGGCTGAGATTGAGCAGAATAGTGGACAAGGAGGACCTAGTGGGTACTGGAGTGGGATGTTAGGTGGAGGGGCATGG | MDTSAQWPQGVGLVKPMEGSRAITERKPRPQKEQALNCPRCNSTNTKFCYYNNYSLTQPRYFCKTCRRYWTEGGSLRNVPVGGGSRKNKRSSSSSSPSPSSKKLPDHLGPPSFSSQSASQNPRIHENLNLAYPASTSDNYHNALNPNPSSTSPHVSAMELLKTGISSRGLSSFMPMPIPDSNSIYPSGFPMQEFKPSLNFSLDGFESGYHHHHHQENSSARLFFPFEELKQAPNTAEIEQNSGQGGPSGYWSGMLGGGAW |
| *CsDof-16* | ATGGATACTGCTCAATGGCCACAGGAGATAGTAGTGAAACCAATGGAAGAGATCATAGTACCAAACACATGTTCAAAGCCAGGGTTAGAGAGGAGGGTTAGGCCTCAGAAAGAACAAGCCTTGAACTGTCCAAGGTGCAATTCAACCAACACAAAGTTCTGTTACTACAAC | MDTAQWPQEIVVKPMEEIIVPNTCSKPGLERRVRPQKEQALNCPRCNSTNTKFCYYN |
| *CsDof-17* | ATGGCAGATCCAAAAACGCCATCTGTTGACAATGAAGCTGTAATATTAAAAACTTCAAAGACTGAAGAAGACCAAAGTGAGACTATTAACTCACAAGAGAAGACCCTAAAGAAACCAGACAAGATACTTCCATGCCCACGCTGTAATAGCATGGACACCAAGTTCTGTTACTACAACAACTACAGTCTCACACAACCAAGATACTTTTGCAAGGGTTGTAGAAGGTATTGGACTGAAGGTGGATCTCTCAGAAACATTCCTGTTGGAGGAGGTTCAAGAAAGAACAAGAGATCTTCATCTTCACCTTCATCAACAACAACTTCATCATCATCTTTATCAAAGAAACTTCCTGATCTGGTTCTTCCACCTCAAAACCCTAAGATCCATGAAGGCCAAGATCTCAACTTGGGTTACCCATCTCATGATTTCAAAACCATCTCTGAACTCATTCAAGTGCCCAATTTTGATGACAACAAGAACAACGCTTCTTGTGCATCCACAACTACTTCATCATCTCACCTCTCAGCTTTGGAGCTTCTCACTGGGATTAACTCAAGGGGGTTGAGTTCTTTCATACCCATGTCGGTGCCGATGATGCCGATATCGGATCAGAACTCATTTTACTCATCTGGGTTTTCTCTGCAGGAATTCAAACCAACCCTAAATTTCTCTCTTGATGGGCTTGGAATTGGAAGTAGTGGGTATGGAAGTCTCCAAGGGGTGCAAGAAACTAGTGGGAGGCCTTTGTTTCCATTTGAAGATCTGAAACTAGTCTCAAGCTCCTCTGATATTGAGCAAAATAGAGAGCAAGGAGATTCAACTGGGTATTGGAATGGAGTCTTAGGTGGTGGTGGTAGTGGTGGTGGATCATGG | MADPKTPSVDNEAVILKTSKTEEDQSETINSQEKTLKKPDKILPCPRCNSMDTKFCYYNNYSLTQPRYFCKGCRRYWTEGGSLRNIPVGGGSRKNKRSSSSPSSTTTSSSSLSKKLPDLVLPPQNPKIHEGQDLNLGYPSHDFKTISELIQVPNFDDNKNNASCASTTTSSSHLSALELLTGINSRGLSSFIPMSVPMMPISDQNSFYSSGFSLQEFKPTLNFSLDGLGIGSSGYGSLQGVQETSGRPLFPFEDLKLVSSSSDIEQNREQGDSTGYWNGVLGGGGSGGGSW |
| *CsDof-18* | ATGTTTGCAGCTAATGATCAAATGCTGCAGTACCCTTCAAGACCATTGATGATAGAAAGAAGCTGGAAGTCCTGCAATGCTGCCGAGATAGCACCAAACTGTCCAAGGTGTGCCTCCACTAACACAAAGTTCTGCTACTACAAC | MFAANDQMLQYPSRPLMIERSWKSCNAAEIAPNCPRCASTNTKFCYYN |
| *CsDof-19* | ATGGAAACCAAGTTTTGTTATTTCAACAACTACAATGTTAATCAACCTAGACACTTCTGCAAAGGCTGCCAGCGGTACTGGACCGCCGGTGGGGCCCTCCGCAACGTCCCCGTCGGTGCTGGCCGCCGCAAGGCCAAGCCGCCCTGCCGGACCTTCGCCGGAGGATTCTCCGATAGTTGCTTCTTTGATGCTTCTTCTGTCCAACAGATTGAATTTGCTGACGGGGTGGTGGTGGAGGAGGAGGAGTGGCGGCGGTTGGCGGCGCACGGTGGTTTCCACCGTGTTTTTCCGGTGAAGAGGCGGAGGAGCACTTCCTCAGGTGGTGGTCAAAGTTGT | METKFCYFNNYNVNQPRHFCKGCQRYWTAGGALRNVPVGAGRRKAKPPCRTFAGGFSDSCFFDASSVQQIEFADGVVVEEEEWRRLAAHGGFHRVFPVKRRRSTSSGGGQSC |
| *CsDof-20* | ATGGCTGATGTCCATGATGGTCATGAAGGCATCAAACTATTTGGTGCAACAATTGCAGTGCAAGTGCGAGAATCAAAGGATGATGAGCCAAACAAAGCTCATGATGATCAAACATTAGAAAAGAGGCCAGACAAGATCATACCATGCCCTAGGTGCAAAAGCATGGAAACAAAGTTTTGTTACTTCAACAACTACAACGTTAATCAACCAAGGTACTTTTGTAAGGGCTGCCAGAGGTATTGGACAGCTGGTGGGGCCCTCCGGAACGTCCCCGTTGGAGCCGGTCGTCGGAAGACTAAGCCGCCGTGCCGGGGAATCTCCGGCTTCTCGGAGAGTTGCTTCTTTGATTCTTCTGAAATGAAACAATTTGATTTTGATGGGGTGGTGGTGGAGTGGCAATTGGCGGCTGCAGCGTCACACGGTGGTTTCCACCGTATTTTTCCGGCGAAGAGGCGGAGGAGCATCGTAGATGGTCAAAGTTGT | MADVHDGHEGIKLFGATIAVQVRESKDDEPNKAHDDQTLEKRPDKIIPCPRCKSMETKFCYFNNYNVNQPRYFCKGCQRYWTAGGALRNVPVGAGRRKTKPPCRGISGFSESCFFDSSEMKQFDFDGVVVEWQLAAAASHGGFHRIFPAKRRRSIVDGQSC |
| *CsDof-21* | ATGAACGGCGGAGATTCGTCAGACACCAAGGATCCAGCCATCAAGCTCTTTGGAAAGACGATTCCTCTCCCGGAATGTCATTTTCCGGGAAAGTCTGGCGAGAAATCCGATGTGGGTTGTCAGATTCCGGACAAGTCCCAAGTTATGGAGTTGTGTAGTGGAATATCAAAAACAGAAGCTGAGGATGTTCATTTCAATGAGTCTGGAAAGCAAGACAAGTCTTTTGGTTCGAAGGATGGTCAGGAGGAGGACAACCCGGCTGAGACTAGCGGTGTCGACCAAGACAAGGCTTTCAAGAAACCTGACAAGGTCATTTCATGTCCTCGGTGCAACAGTTTTGACACTAAGTTCTGCTACTTCAATAACTACAATGTCAATCAGCCTCGACATTTTTGCAAGAATTGCCAAAGATATTGGACAGCTGGTGGAACAATGAGAAATGTTCCTGTTGGTGCTGGCAGGCGAAAGAACAAGCACTTCTCTTCTCAATATCGTCAGATAGTGGTGTCCCCCGATTCAGTACCAGCAACAAGAATCGAAATCCCTGACTCTGTCAATCAACAACTTTTCTCTTGTGGTGAATCTCAGAGCACGTTGAATCCTTTAACTAGCAGTGGAACTGTACTAAAATTTGGTCCCGAAGGACGTTTATGTGAATCCATGACAACTGTGCTTCATCTCAAAGATCAGAAAAGCTGTGCTTCTGATTTTGGTTCGTCCAGAGATAAAAGAGAGGACCCTCCCTCATGTGGTTCTTCAATTACAGCGTCCAGTATCCAGGAAAATCAGATACCCGAAAAGGTTAACGAGCTTTCTCAGCCAAATCCCTTGCAATGTTATCCAGTTCCTCCTTGGGTTTCCCCTTGGAATCAAGCATGGAGTGATGGAGCTTCCATGGTGGCATCTCGGTGCTCTTCTGAACTGGTTTCTGCACCAGTTGATGGTAATCCAAACCCAATTCAATGGTGCTCGACACCAATGCTTGCAGTTCCTGGATTTTGCCCGCCTAGCATTCCTGTACAGTTTGTACCTGCATCATATTGGGGTTGCTTGCCTGTTTGGACAACAGGAACAAGACATATACCAATGGTGGGAGTGGATGGTGGCCAATCTCCATCATTATCCACTAGCAACAACTTACCAACGTTAGGAAAGCATTCCCGGGATGCAAATTTGATGGATGAGGAGTCAGATAAGTGTGTTTTAGTTCCCAAGACACTGAGAATTGATCACTCTGATGAGGCTTCTAAGAGTTCTATCTGGGGCACATTAGGTTTTAAGTCTAACAAGAAAGAAACTGAAGAATCAAGATGTGGGATTTTCAAACCATTCAAACCTAGCGGCAGTGAATGCAAGGGCCATCCATCAGATACTACTCAGGTGTTAGAAGCCAACCCTGCAGCTCTTTCTCGCTCCCATACATTTCAAGAGAGCACC | MNGGDSSDTKDPAIKLFGKTIPLPECHFPGKSGEKSDVGCQIPDKSQVMELCSGISKTEAEDVHFNESGKQDKSFGSKDGQEEDNPAETSGVDQDKAFKKPDKVISCPRCNSFDTKFCYFNNYNVNQPRHFCKNCQRYWTAGGTMRNVPVGAGRRKNKHFSSQYRQIVVSPDSVPATRIEIPDSVNQQLFSCGESQSTLNPLTSSGTVLKFGPEGRLCESMTTVLHLKDQKSCASDFGSSRDKREDPPSCGSSITASSIQENQIPEKVNELSQPNPLQCYPVPPWVSPWNQAWSDGASMVASRCSSELVSAPVDGNPNPIQWCSTPMLAVPGFCPPSIPVQFVPASYWGCLPVWTTGTRHIPMVGVDGGQSPSLSTSNNLPTLGKHSRDANLMDEESDKCVLVPKTLRIDHSDEASKSSIWGTLGFKSNKKETEESRCGIFKPFKPSGSECKGHPSDTTQVLEANPAALSRSHTFQEST |
| *CsDof-22* | ATGAGGAATGTTCCAGTAGGTTCTGGTCGTCGCAAAAACAAAAACGCTTCCGCTTCACATTATCGTCATATCTTGGTTGCTCAAGCAGATGTTGCTAATGGAACTGTCCTTAATTTTGGCTCGGACTCACCACTTTGTGAATCCATGGATTCTGCATTGACCCTAGCTGGGAAATCACAGAAGATTTCTGTTTCGTGTGGAGGAAGAGAATATGGGGATGAGCGGTCAAGTAGGTCTTCAATTACAGGTTCAGATTCAGCAGAAAGGGGAGGGAATTCGATTTTGCAAGAATCGGTTACGACCAATTTTCAGAACTTGAATCCTCAAGTACCGTGCTTCTCGGGTCCTCCTTGGCCTTGTCAATGGAACTCACCCCAATGGAGGACTCCAATGCCTCAACCGGCTTTTGGCCCTTCTGGCTTTCCAATTGCAGTTTACCCAGCACCACCTTATTGGGGTTGTGCTTTACCTGGATCTTGGAATTTTCCATGGATTTCGCCTGACCCTGCTACATGCTCAAGTCCTAATTCTCCAATCCTGGGGAAGCATTCCAGGGATTGGGATGTTCTTAAACCATCAAGCTCGGAGAAAGAAGAGGCCTTGAAAGAAAACAATTTGGAGAGATGTGTTTGGATTCCAAAGACATTGAGGATCGATGATCCAAGTCAAGCAGCAAAAAGCTCTATATGGTCAACACTCGGGATTAAGAATGAAAAGAAGGATTCAATCAACACGGGAGGTCTGTTTAAGGCATTCCAATCAAAAGGAGATGAGAAAAAACACATAGACGAAACATCTTTGGTGTTGCAAGCCAACCCTGCAGCCTTGTCAAGGTCACTCAACTTCCAAGAGAGTGGC | MRNVPVGSGRRKNKNASASHYRHILVAQADVANGTVLNFGSDSPLCESMDSALTLAGKSQKISVSCGGREYGDERSSRSSITGSDSAERGGNSILQESVTTNFQNLNPQVPCFSGPPWPCQWNSPQWRTPMPQPAFGPSGFPIAVYPAPPYWGCALPGSWNFPWISPDPATCSSPNSPILGKHSRDWDVLKPSSSEKEEALKENNLERCVWIPKTLRIDDPSQAAKSSIWSTLGIKNEKKDSINTGGLFKAFQSKGDEKKHIDETSLVLQANPAALSRSLNFQESG |
| *CsDof-23* | ATGTCGGAATCAAAAGACCCTGCGATTAAGCTCTTCGGAAAGACGATTCCCTTGCCGGATATTCCGCCTCCGCCCTCCGCCGGACCTCCTCCTCTTCTGTCTGATTCTGAAAGTCTCAATCTTGATCGTTCTTCGTCGCCGGAGGATGACAAGTCGAGCAGAGTCGGAGAAGATCATTACCCTGATAATAAGGCCCCATCTGGTGGAAGCCTGAGTGATACTAAACAGGTGGATGGAGCCCGGCCTTTTCCTCCGGAGGAGATTGCAGATCCAACTACAGTGGTAAATGAGAACCCAAAAACACCGTCCGTTGACAATGAAGCTGCAACATTAAAAGCTTCAAAGACTGAAGAAGAACAAAGTGAGACCAGTAACTCACAGGAGAAGACCCTGAAGAAACCGGACAAGATTCTTCCATGCCCTCGCTGCAATAGCATGGACACCAAGTTTTGTTACTACAACAACTACAATGTCAACCAGCCCCGGCACTTCTGTAAGAATTGCCAGAGATACTGGACTGCTGGTGGGACCATGAGGAATGTGCCTGTTGGTGCTGGTCGTCGGAAGAACAAGAACTCAGCTTCTCACTACCGTCACATAGCTGTATCAGAAGCTCTTCAGAATGCCCGAGCAGATCTTCCAAATGGAATCCACCATTCCACTCTAAAGCCCAACGGCACTGTCCTCACTTTTGGCTCATCAGACACACCCCTTTGTGAGTCAATGGCCTCAGTTTTGAACATTGCAGAGAAAACGATGAGGAACTGTGCCCAAACTGGGTTTCGTAAACCAGAAAAACTAATAATTCCAGTTTCTTACGTAGGTGGAGAGAACGGGGATGATCACTCAAGTGGATCTTCAGTCACTGCTGCAAATTCAAAGGATGATGTTGGTAGTAAAACAGGGCTACAAGATCCAGTAATGCAGAATTGTCATGGCTTCCCACCTCAAGTACCATGCTTTCCTGGCACTCCGTGGCCATACCCATGGAATTCAGCTCAATGGAGTGGTCCGGTACCTGTACCCGCTTTCTGCCCCCCAGGCTTTCCTATGCCATTCTATCCTCCACCGTATTGGGGTTGTACCATACCAGGAACTTGGAATGTCCCTTGGGTGACACCGCCTAGTTCTTCTCCAAACCACATAGCTCCTAGTTCTGGTCCTAATTCCCCAACCTTGGGGAAGCATTCTAGGGATGAAAACATGCTTAAGCAAACCAAATCCGGGGAAGAGGACCCACAGAGAGAGAATAATCCTGAGAGGTCCCTATGGATTCCAAAAACTTTGAGAATTGATGACCCAGGGGAAGCTGCAAAAAGTTCTATATGGGCAACATTGGGAATTAAAAATGATAAAGTCGATTCAGTTAGTGGGGGAGGTCTGTTTAAGCCCTTCCAACCGAAAGGCGATGACAAGAGTCATGTCTCTGAAACCTCTCCAGTATTACAAGCCAATCCTGCAGCGTTGTCTAGGTCGCTTGACTTCCATGAAAGCTCA | MSESKDPAIKLFGKTIPLPDIPPPPSAGPPPLLSDSESLNLDRSSSPEDDKSSRVGEDHYPDNKAPSGGSLSDTKQVDGARPFPPEEIADPTTVVNENPKTPSVDNEAATLKASKTEEEQSETSNSQEKTLKKPDKILPCPRCNSMDTKFCYYNNYNVNQPRHFCKNCQRYWTAGGTMRNVPVGAGRRKNKNSASHYRHIAVSEALQNARADLPNGIHHSTLKPNGTVLTFGSSDTPLCESMASVLNIAEKTMRNCAQTGFRKPEKLIIPVSYVGGENGDDHSSGSSVTAANSKDDVGSKTGLQDPVMQNCHGFPPQVPCFPGTPWPYPWNSAQWSGPVPVPAFCPPGFPMPFYPPPYWGCTIPGTWNVPWVTPPSSSPNHIAPSSGPNSPTLGKHSRDENMLKQTKSGEEDPQRENNPERSLWIPKTLRIDDPGEAAKSSIWATLGIKNDKVDSVSGGGLFKPFQPKGDDKSHVSETSPVLQANPAALSRSLDFHESS |
| *CsDof-24* | ATGTCGGAATCAAAAGACCCTGCGATTAAGCTCTTCGGAAAGACGATTCCTTTGCCGGAGATTCCGCCGGCGCCCGCCATCGCAGTCTCCGGCGGCGGAGCTCCCTCTCCCTCTCCTCCTCCCCCTCCTCCTCCTGTTGATGATCCTTGTTCGCCTGACTCGAACAGAGTCGAAGAAGATCAAGAACCTGATAAGGCCCCGGCTAATCGAAGTGATACTAATCGGGAGGATGGAGCCCAGCCTATTACTTCAGAAGAGATTGCTGATCCAAAAACGCCATCTGTTGACAATGAAGCTGTAATATTAAAAACTTCAAAGACCGAAGAAGACCAAAGTGAAACTATTAACTCACAAGAGAAGACCCTAAAGAAACCAGACAAGATACTTCCATGCCCACGCTGTAATAGCATGGACACCAAGTTCTGTTACTACAACAACTACAATGTCAACCAGCCCCGGCACTTCTGTAAGAACTGCCAGAGATACTGGACAGCTGGTGGGACCATGAGGAATGTTCCTGTGGGTGCTGGTCGCCGGAAGAACAAAAACTCAGCCTCTCACTACCGTCACATAACCGTATCAGAAGCTCTTCAGAGCGATCGAGCAGAGATTCCAAATGGAATCCACCATCCCACTCTGAAGCCCAATGGCACTATCCTCACTTTTGGTTCGGATGCTCCACTTTGCGAGTCAATGGCTTCAGTTTTAAACATTGTGGAAAAAACGTTGCCAAATCATGCTCAAAATGGGTTTTGTAAACCGGAGGAACTAAGAATCTCGGTTTCTTATAGTGGTGGAGAGAATGGGGATGATCACTCTAGTGGATCCGCAGCCACTGGTGCAAATTTGAAAGATGATGTTGGTAAAAATGGGCAACAAGATACGAAAATCAAGAATTGTCAGAGCTTCTTTCCTCAGGTACCGTGTTTTCCCGGGGCTCCGTGGTCGTACCCTTGGAATTCTGCTCAATGGAGATCCCCGGTACCCCCACCTGCTTTTTGCCCACCAGGCTTTCCAATGCCATTCTATCCTCCACCGCCTTATTGGGGTTGTACTGTACCAGGCATTTGGAACGTCTCTTGGATAACACCGCCAAACCACACAGCTCCTAGTTCTGGTCCTAATTCCCCGACTTTGGGGAAGCATTCTAGGGATGAGAACGCGCTTAACCAAACCAATTCTGGGGAAGATGAGCCACAGAAAGAGAATAATCCTGAGAAGTGCCTCTGGATTCCAAAAACTTTGAGAATTGATGATCCTGGTGAAGCTGCGAGGAGTTCTATATGGGCAACATTGGGAATTAAAAACGATAAAGTTGATTCAATTAGCGGGGGAGGTCTCTTTAAGGCCTTCCAATCGAAGGGTGGTGATGAGAAGCATCACATCTCTGAAACCTCTCCGGTATTACAAGCCAATCCAGCAGCATTGTCTAGGTCATTCAACTTCCATGAGAGTTCA | MSESKDPAIKLFGKTIPLPEIPPAPAIAVSGGGAPSPSPPPPPPPVDDPCSPDSNRVEEDQEPDKAPANRSDTNREDGAQPITSEEIADPKTPSVDNEAVILKTSKTEEDQSETINSQEKTLKKPDKILPCPRCNSMDTKFCYYNNYNVNQPRHFCKNCQRYWTAGGTMRNVPVGAGRRKNKNSASHYRHITVSEALQSDRAEIPNGIHHPTLKPNGTILTFGSDAPLCESMASVLNIVEKTLPNHAQNGFCKPEELRISVSYSGGENGDDHSSGSAATGANLKDDVGKNGQQDTKIKNCQSFFPQVPCFPGAPWSYPWNSAQWRSPVPPPAFCPPGFPMPFYPPPPYWGCTVPGIWNVSWITPPNHTAPSSGPNSPTLGKHSRDENALNQTNSGEDEPQKENNPEKCLWIPKTLRIDDPGEAARSSIWATLGIKNDKVDSISGGGLFKAFQSKGGDEKHHISETSPVLQANPAALSRSFNFHESS |
| *CsDof-25* | ATGAGGAACGTGCCCATAGGAGCTGGTCGGCGCAAGAACAAGAACTCATCCGTGCATTGTCGCCACATCACCATCTCTGAAGCTCTACATGCAACTGCAAATGGATTTCAGAACCCTACGCTCAAAAGCAATGGCACGGTTCTCTCTTTTGGCCCAGAAGCACCACTTTGTGAATCCATGGCTTCTGTCTTAAATCTTGCGGAGAAACAAAAGGCTCCTAATGGAATCCCCAATGGGTTTTATAAAGTTGAACAAAATGGCGATGATTGTTCTAGCGGATCATCTGTCACAACTTCAAATTCGACAGAGGAAGGAGTGAGAAACGGGATTCAAGAGCCAATGATGGGGAATTTAAATGGCTTCACTTCCCAAATCCCTTGCCTCCCTGGCATTCCATGGCCTTATTGGAATTCAGCTGCTCCTATACCGGCTGTTTGCTTGTCTGGATATCCAATGCCATTCTATGCCACCCCCTATTGGACAGTTCCATGGTTGCCTTCGCCATCTCCTACAACAAACCAAAACGTGCCATGCTCTGCTCCAAACCAAAATTCACCGACTCTAGGGAAACATTCAAGAGAAGGGGACTTGATTAAGAAGAATTCAGAGACATCCATTTTGATTCCAAAAACTATAAGGATTGATGACCCTGATGAAGCTGCAAGGAGTTCTATATGGGCAACACTTGGGATTAAGAACGATTCCTCAATGATGAGCAGAGGAGGCCTTTTCAAGGCCTTCCAACCAAAGGGTGGCGAAAAGAATCACACACCAGAAACCAATTTAGCGCTGTGGGCTAACCCTGCAGCCTTGTCTAGGTCGCTCAGCTTCCAAGAACGGTCC | MRNVPIGAGRRKNKNSSVHCRHITISEALHATANGFQNPTLKSNGTVLSFGPEAPLCESMASVLNLAEKQKAPNGIPNGFYKVEQNGDDCSSGSSVTTSNSTEEGVRNGIQEPMMGNLNGFTSQIPCLPGIPWPYWNSAAPIPAVCLSGYPMPFYATPYWTVPWLPSPSPTTNQNVPCSAPNQNSPTLGKHSREGDLIKKNSETSILIPKTIRIDDPDEAARSSIWATLGIKNDSSMMSRGGLFKAFQPKGGEKNHTPETNLALWANPAALSRSLSFQERS |
| *CsDof-26* | ATGATGGTGACGGCGAGGGACCCAGCGATCAAGCTGTTCGGGAAGGAGATTCCGCTGCCGAGAAACGGGCTGACTTGTTTGGAAGATGGGAGGTCTGATCATGATCTTAGTTCTCTTGTGGATGCCTGTTTGGAAGTCGAGAAAGTAAGCAAAACACGGGAGGAAGAAGAAAACGAACCAGAAGCTGATCACAAGTATCCACTAGCAGAAAAACCTCCAACTACAGAGGACCCTGCAAATCAAGAAACATTGCTGGAGTCTGATGAAAACCCTAAAACACCCTCGTCTGGCGATGAAAAACCTCCAAAAACTGAAAATGATGGAAGTGATACAAATAGCCCCGAACAGAAAACCCTGAAAAAGCCAGATAAAATCCTCCCATGCCCGCGTTGTAAAAGCATGGACACGAAATTCTGTTACTACAACAATTACAACGTCAATCAGCCCCGCCATTTCTGCAAGAGCTGTCAGAGGTACTGGACTGCTGGCGGCACCATGAGGAACGTGCCTGTAGGAGCTGGAAGACGCAAGAACAAGAATTCTGCCTCCTCGCATTGTCGCCACATCACAATATCCAAGGCTCTCCACTCTGCTGAAATTGATACCCCCAATGGAATTCACCGTCCTGCGTTCAAAACCAATGGCACGGTCCTCTCATTTGGCACAGAAGCACCAATTTTTGAATCCGTGGCTTGTGTCTTGAATCTTGCAGAGAAAAAGGCTCCTGTTGGTGGAGAAAAGGGGGATGATTGCTCTAGTGGTTCATCTGTCACAACTTCAAATTCATTGGAAGGAGGAGAGAGGAATGGGATTCAAGAACGCTTTGTTCCTCAAATTCCATGCCTCCCTGGGGTTCCATGCCCCCCTGGGGTTCCATACCCTTATCCTTGGAATACCTCGATTCCTGTACCGGCCATTTGCCCTTCTGTAATCCCCATGCCATTCTACCCTGCCCCATATTGCCCCGCCCCATATTGGAATTGCAGTGTACCGGATGCTTGGAGCATTCCATATCTACCAACTCTAGGGAAGCGATCAAGAGACGGGGACTTGGTAAAACCGAGCAATTCAGATGAAAAAGAGCCTAAAAATTCAGAGAGGTCTATTTTGATTCCGAAAACTTTGAGGATTGATGACCCTGACGAAGCTGCAAGGAGCTCTATATGGACAACGCTTGGGATTAAGAACGATTCCATTAGCGGGAAAGGACTTTTTAAGGCTTTTGAACCAAAGGGCAAAGAAAAGAATCACATAGCCGAAACCCTACCATCAATGCAAGCTAACCCTGCAGCTTTCTCTAGGTCCCTCAGCTTTCAAGAGGCCGCC | MMVTARDPAIKLFGKEIPLPRNGLTCLEDGRSDHDLSSLVDACLEVEKVSKTREEEENEPEADHKYPLAEKPPTTEDPANQETLLESDENPKTPSSGDEKPPKTENDGSDTNSPEQKTLKKPDKILPCPRCKSMDTKFCYYNNYNVNQPRHFCKSCQRYWTAGGTMRNVPVGAGRRKNKNSASSHCRHITISKALHSAEIDTPNGIHRPAFKTNGTVLSFGTEAPIFESVACVLNLAEKKAPVGGEKGDDCSSGSSVTTSNSLEGGERNGIQERFVPQIPCLPGVPCPPGVPYPYPWNTSIPVPAICPSVIPMPFYPAPYCPAPYWNCSVPDAWSIPYLPTLGKRSRDGDLVKPSNSDEKEPKNSERSILIPKTLRIDDPDEAARSSIWTTLGIKNDSISGKGLFKAFEPKGKEKNHIAETLPSMQANPAAFSRSLSFQEAA |
| *CsDof-27* | ATGTCTTGTTGTCCATCAGAAAGAGAAGTGATTGAAGAAGCAAGATTATACTCAGAAGAACTGTCCGGTTGTTGTTGCTCATCATCATCATCATCATCATTAAGAAGTAGATCAGTGCTATTTGGGAACAATTTTAGCAGCTGCAATGGTGTAGAAGAAGCAGTGCCGAGAGAGAGGGTGGATTGTGTAGTGATCGGAGCAGGAGTGGTGGGAATAGCTGTAGCCAGAGATCTCTCTCTGAAACTAAGAAGACAAGTTCTGGTAATCGATTCTGGTCCTACGTTCGGGACAGGAACTAGCTCTCGCAACAGCGAAGTCATCCATGCCGGCATTTACTACCCTCCCAATTCCCTCAAGGCACTGTTTTGTGTTAAAGGAAGAGAATTGCTGTACAAGTATTGCAAAGAACATGATATTCCTCATAAACAGATTTGTAAACTCATAGTTGCTACTGGATCTTCAGAGATTCCAAAGTTGAATAGTCTGATGAATCTTGGGATTGAAAATGGGGTTGACGGTCTAAGGATGATGGAAGGTTATGAAGCCATGAGAATTGAGCCTGAATTGCAATGTATTAAAGCTTTATCATCACCCGTGTCAGGAATAGTTGATACCCATTCCCTAATGTTATCGTTAGTGGGGGAAGCTGAGAGTTACGGAACGACGTTCTCCTACAACACTACTGTGATTGGTGGTCATCGTGAAGGAAATACTATTTGCCTTCATATTTCTAATAGCAAGGCTCTTGAAAATTGGGATGGGAAATCTCCGTTGCAACCAGAGCTTGTACTTGTTCCTAACTTTGTAGTGAACTCTGCAGGCTTGAGTGCTTCGTCCCTTGCAAAGCGATTCGATGGCATA | MPAWMTSLLRELVPVPNVGPESITRTCLLSFRERSLATAIPTTPAPITTQSTLSLGTASSTPLQLLKLFPNSTDLLLNDDDDDDEQQQPDSSSEYNLASSITSLSDGQQDIKKYSESELAETRQEDHTSHPTAEEIKYPATSCENPKTPSIDKEPVSPEESKNDEGSETSVSQEKTLKKPDKILPCPRCNSMDTKFCYYNNYNVN |
| *CsDof-28* | ATGTCGGAAGTGAAAGACCATGCGATCAAGTTGTTTGGCAAGACGATTCCATTGCTTCTCAACGAGCAAGTCTCAGTTGTTACAACACCACTAAAAACAACAAAAACCCACGTTGATTCTGATGAACATCATGATGATAACCATCAACAACACCACTCTACCTCCCATCAAAATCTTCTTTCTTCAGCCTCTTGTTCGACGCAAAGTCAAAACAGAGTTGAAGATGTGCAAGAAAACAACCAGAAACCTTCGCAAATTGAAGTCACCGAGACTAGACAGGAAGATCCAGCAGCAACACCATGTGAAAACCCGAAAACACCATCTGTCGAAAAAGAGCATAGTTCATCCATAGTTTCCAAGAACGACGAACAGAGCGAGACTAGCAATAACTCACAAGAAAAAACACTGAAAAAGCCTGACAAAATACTTCCATGTCCCCGCTGCAATAGCATGGACACCAAG | MSEVKDHAIKLFGKTIPLLLNEQVSVVTTPLKTTKTHVDSDEHHDDNHQQHHSTSHQNLLSSASCSTQSQNRVEDVQENNQKPSQIEVTETRQEDPAATPCENPKTPSVEKEHSSSIVSKNDEQSETSNNSQEKTLKKPDKILPCPRCNSMDTK |
| *CsDof-29* | ATGAGGGACCCGGCGATCAAGCTTTTTGGGATGGAGATTACGCTGCCGGAGACCGGAAAGATTCTTGTCGGCGAGGATTGTGGGGTTGAAGATGGGAGGTCTGATGGTGATCGTAGTTCGGTGATGAGTTCGTGTTTGGAGGACGAGAAAATGGACAAGAAAGTTAAGAGAACAGGGGACGAAGAGGAAGAGGAAAAAAAAGTAGAAGCTGATGATGACAAGGATTCACAAGCAAGAAAGCCCGATGAGAGTAAACAACGTGGTAAATCTCCACCTTCAGAAACATTGCCCGAGTCTGAGGATAACCCTAAGACACCGTCTCTCGATGAAGAAAATGCAAGTGCAAAACCTCAAAAGAATGAAAATGACCAAAATGATGCAAACAACACACAACAAAAAACCCTAAAGAAGCCTGACAAAATTCTTCCATGCCCCCGCTGTAATAGCATGGACACAAAGTTCTGTTACTACAAC | MRDPAIKLFGMEITLPETGKILVGEDCGVEDGRSDGDRSSVMSSCLEDEKMDKKVKRTGDEEEEEKKVEADDDKDSQARKPDESKQRGKSPPSETLPESEDNPKTPSLDEENASAKPQKNENDQNDANNTQQKTLKKPDKILPCPRCNSMDTKFCYYN |

**Table S3** Gene codes and amino acid sequences of Dof TFs from *Arabidopsis*

| Gene code. | amino acid sequences |
| --- | --- |
| AT1G07640.1 | MGGSMAERARQANIPPLAGPLKCPRCDSSNTKFCYYNNYNLTQPRHFCKGCRRYWTQGGALRNVPVGGGCRRNNKKGKNGNLKSSSSSSKQSSSVNAQSPSSGQLRTNHQFPFSPTLYNLTQLGGIGLNLAATNGNNQAHQIGSSLMMSDLGFLHGRNTSTPMTGNIHENNNNNNNENNLMASVGSLSPFALFDPTTGLYAFQNDGNIGNNVGISGSSTSMVDSRVYQTPPVKMEEQPNLANLSRPVSGLTSPGNQTNQYFWPGSDFSGPSNDLL |
| AT1G07640.2 | MPTNSNHQHHLQHQLNENGSIISGHGLVLSHQLPPLQANPNPNHHHVATSAGLPSRMGGSMAERARQANIPPLAGPLKCPRCDSSNTKFCYYNNYNLTQPRHFCKGCRRYWTQGGALRNVPVGGGCRRNNKKGKNGNLKSSSSSSKQSSSVNAQSPSSGQLRTNHQFPFSPTLYNLTQLGGIGLNLAATNGNNQAHQIGSSLMMSDLGFLHGRNTSTPMTGNIHENNNNNNNENNLMASVGSLSPFALFDPTTGLYAFQNDGNIGNNVGISGSSTSMVDSRVYQTPPVKMEEQPNLANLSRPVSGLTSPGNQTNQYFWPGSDFSGPSNDLL |
| AT1G07640.3 | MAFPSNWSQPTNSNHQHHLQHQLNENGSIISGHGLVLSHQLPPLQANPNPNHHHVATSAGLPSRMGGSMAERARQANIPPLAGPLKCPRCDSSNTKFCYYNNYNLTQPRHFCKGCRRYWTQGGALRNVPVGGGCRRNNKKGKNGNLKSSSSSSKQSSSVNAQSPSSGQLRTNHQFPFSPTLYNLTQLGGIGLNLAATNGNNQAHQIGSSLMMSDLGFLHGRNTSTPMTGNIHENNNNNNNENNLMASVGSLSPFALFDPTTGLYAFQNDGNIGNNVGISGSSTSMVDSRVYQTPPVKMEEQPNLANLSRPVSGLTSPGNQTNQYFWPGSDFSGPSNDLL |
| AT1G21340.1 | MLPYIGHNSYQQHQFPLPEMEIPEKWKLSYEQEAITAPACPRCASSNTKFCYYNNYSLSQPRYFCKGCRRYWTKGGSLRNIPVGGGCRKRSRSRQNSHKRFGRNENRPDGLINQDDGFQSSPPGSDIDLAAVFAQYVTDRSPSSTDNTTGSDQDSPITTTTHALESLSWDICQETDVDLGFYGEFNNLTQKTKEDQEVFGQFLQEDREEIFEFQGLLDDKEIQEILECSFSEEPDQLVSQGSFMINGDNWSSTDLTRFGI |
| AT1G26790.1 | MWLSHLFMSLSKLTCNFSIFSVFMACGSIGMSQVRDTPVKLFGWTITPVSHDPYSSSSHVLPDSSSSSSSSSLSLRPHMMNNQSVTDNTSLKLSSNLNNESKETSENSDDQHSEITTITSEEEKTTELKKPDKILPCPRCNSADTKFCYYNNYNVNQPRHFCRKCQRYWTAGGSMRIVPVGSGRRKNKGWVSSDQYLHITSEDTDNYNSSSTKILSFESSDSLVTERPKHQSNEVKINAEPVSQEPNNFQGLLPPQASPVSPPWPYQYPPNPSFYHMPVYWGCAIPVWSTLDTSTCLGKRTRDETSHETVKESKNAFERTSLLLESQSIKNETSMATNNHVWYPVPMTREKTQEFSFFSNGAETKSSNNRFVPETYLNLQANPAAMARSMNFRESI |
| AT1G28310.1 | MQSKNMIVASSHQQQQQQQPQQPQPQLKCPRCDSSNTKFCYYNNYSLSQPRHFCKACKRYWTRGGTLRNVPVGGSYRKNKRVKRPSTATTTTASTVSTTNSSSPNNPHQISHFSSMNHHPLFYGLSDHMSSCNNNLPMIPSRFSDSSKTCSSSGLESEFLSSGFSSLSALGLGLPHQMSHDHTINGSFINNSTTNKPFLLSGLFGSSMSSSSTLLQHPHKPMNNGGDMLGQSHLQTLASLQDLHVGGNNEDMKYKEGKLDQISGNINGFMSSSSSLDPSNYNNMWNNASVVNGAWLDPTNNNVGSSLTSLI |
| AT1G28310.2 | MPIISSPNTNPLASMQSKNMIVASSHQQQQQQQPQQPQPQLKCPRCDSSNTKFCYYNNYSLSQPRHFCKACKRYWTRGGTLRNVPVGGSYRKNKRVKRPSTATTTTASTVSTTNSSSPNNPHQISHFSSMNHHPLFYGLSDHMSSCNNNLPMIPSRFSDSSKTCSSSGLESEFLSSGFSSLSALGLGLPHQMSHDHTINGSFINNSTTNKPFLLSGLFGSSMSSSSTLLQHPHKPMNNGGDMLGQSHLQTLASLQDLHVGGNNEDMKYKEGKLDQISGNINGFMSSSSSLDPSNYNNMWNNASVVNGAWLDPTNNNVGSS  LTSLI |
| AT1G29160.1 | MATQDSQGIKLFGKTITFNANITQTIKKEEQQQQQQPELQATTAVRSPSSDLTAEKRPDKIIPCPRCKSMETKFCYFNNYNVNQPRHFCKGCQRYWTAGGALRNVPVGAGRRKSKPPGRVGGFAELLGAATGAVDQVELDALLVEEWRAATASHGGFRHDFPVKRLRCYTDGQSC |
| AT1G47655.1 | MPSEPNQTRPTRVQPSTAAYPPPNLAEPLPCPRCNSTTTKFCYYNNYNLAQPRYYCKSCRRYWTQGGTLRDVPVGGGTRRSSSKRHRSFSTTATSSSSSSSVITTTTQEPATTEASQTKVTNLISGHGSFASLLGLGSGNGGLDYGFGYGYGLEEMSIGYLGDSSVGEIPVVDGCGGDTWQIGEIEGKSGGDSLIWPGLEISMQTNDVK |
| AT1G51700.1 | MQDLTSAAAYYHQSMMMTTAKQNQPELPEQEQLKCPRCDSPNTKFCYYNNYNLSQPRHFCKNCRRYWTKGGALRNIPVGGGTRKSNKRSGSSPSSNLKNQTVAEKPDHHGSGSEEKEERVSGQEMNPTRMLYGLPVGDPNGASFSSLLASNMQMGGLVYESGSRWLPGMDLGLGSVRRSDDTWTDLAMNRMEKN |
| AT1G64620.1 | MDTAKWPQEFVVKPMNEIVTNTCLKQQSNPPSPATPVERKARPEKDQALNCPRCNSLNTKFCYYNNYSLTQPRYFCKDCRRYWTAGGSLRNIPVGGGVRKNKRSSSNSSSSSPSSSSSSKKPLFANNNTPTPPLPHLNPKIGEAAATKVQDLTFSQGFGNAHEVKDLNLAFSQGFGIGHNHHSSIPEFLQVVPSSSMKNNPLVSTSSSLELLGISSSSASSNSRPAFMSYPNVHDSSVYTASGFGLSYPQFQEFMRPALGFSLDGGDPLRQEEGSSGTNNGRPLLPFESLLKLPVSSSSTNSGGNGNLKENNDEHSDHEHEKEEGEADQSVGFWSGMLSAGASAAASGGSWQ |
| AT1G69570.1 | MSKSRDTEIKLFGRTITSLLDVNCYDPSSLSPVHDVSSDPSKEDSSSSSSSCSPTIGPIRVPVKKSEQESNKFKDPYILSDLNEPPKAVSEISSPRSSKNNCDQQSEITTTTTTSTTSGEKSTALKKPDKLIPCPRCESANTKFCYYNNYNVNQPRYFCRNCQRYWTAGGSMRNVPVGSGRRKNKGWPSSNHYLQVTSEDCDNNNSGTILSFGSSESSVTETGKHQSGDTAKISADSVSQENKSYQGFLPPQVMLPNNSSPWPYQWSPTGPNASFYPVPFYWGCTVPIYPTSETSSCLGKRSRDQTEGRINDTNTTITTTRARLVSESLRMNIEASKSAVWSKLPTKPEKKTQGFSLFNGFDTKGNSNRSSLVSETSHSLQANPAAMSRAMNFRESMQQ |
| AT2G28510.1 | MDPEQEISNETLETILVSSTKGSNNNNKKMEEEMKKKVSRGELGGEAQNCPRCESPNTKFCYYNNYSLSQPRYFCKSCRRYWTKGGTLRNVPVGGGCRRNKRSSSSAFSKNNNNKSINFHTDPLQNPLITGMPPSSFGYDHSIDLNLAFATLQKHHLSSQATTPSFGFGGDLSIYGNSTNDVGIFGGQNGTYNNSLCYGFMSGNGNNNQNEIKMASTLGMSLEGNERKQENVNNNNNNSENPSKVFWGFPWQMTGDSAGVVPEIDPGRESWNGMVSSWNNGLLNTPLV |
| AT2G28810.1 | MVFSSVSSFLDPPINWPQSANPNNHPHHHQLQENGSLVSGHHQVLSHHFPQNPNPNHHHVETAAATTVDPSSLNGQAAERARLAKNSQPPEGALKCPRCDSANTKFCYFNNYNLTQPRHFCKACRRYWTRGGALRNVPVGGGCRRNKKGKSGNSKSSSSSQNKQSTSMVNATSPTNTSNVQLQTNSQFPFLPTLQNLTQLGGIGLNLAAINGNNGGNGNTSSSFLNDLGFFHGGNTSGPVMGNNNENNLMTSLGSSSHFALFDRTMGLYNFPNEVNMGLSSIGATRVSQTAQVKMEDNHLGNISRPVSGLTSPGNQSNQYWTGQGLPGSSSNDHHHQHLM |
| AT2G34140.1 | MATQDSQGIKLFGKTIAFNTRTIKNEEETHPPEQEATIAVRSSSSSDLTAEKRPDKIIACPRCKSMETKFCYFNNYNVNQPRHFCKGCHRYWTAGGALRNVPVGAGRRKSKPPGRVVVGMLGDGNGVRQVELINGLLVEEWQHAAAAAHGSFRHDFPMKRLRCYSDGQSC |
| AT2G37590.1 | MVFSSIQAYLDSSNWQQAPPSNYNHDGTGASANGGHVLRPQLQPQQQPQQQPHPNGSGGGGGGGGGSIRAGSMVDRARQANVALPEAALKCPRCESTNTKFCYFNNYSLTQPRHFCKTCRRYWTRGGALRNVPVGGGCRRNRRTKSNSNNNNNSTATSNNTSFSSGNASTISTILSSHYGGNQESILSQILSPARLMNPTYNHLGDLTSNTKTDNNMSLLNYGGLSQDLRSIHMGASGGSLMSCVDEWRSASYHQQSSMGGGNLEDSSNPNPSANGFYSFESPRITSASISSALASQFSSVKVEDNPYKWVNVNGNCSSWNDLSAFGSSR |
| AT2G46590.1 | MMNVKPMEQIMIPNNNTHQPNTTSNARPNTILTSNGVSTAGATVSGVSNNNNNTAVVAERKARPQEKLNCPRCNSTNTKFCYYNNYSLTQPRYFCKGCRRYWTEGGSLRNVPVGGSSRKNKRSSSSSSSNILQTIPSSLPDLNPPILFSNQIHNKSKGSSQDLNLLSFPVMQDQHHHHVHMSQFLQMPKMEGNGNITHQQQPSSSSSVYGSSSSPVSALELLRTGVNVSSRSGINSSFMPSGSMMDSNTVLYTSSGFPTMVDYKPSNLSFSTDHQGLGHNSNNRSEALHSDHHQQGRVLFPFGDQMKELSSSITQEVDHDDNQQQKSHGNNNNNNNSSPNNGYWSGMFSTTGGGSSW |
| AT2G46590.2 | MDATKWTQGFQEMMNVKPMEQIMIPNNNTHQPNTTSNARPNTILTSNGVSTAGATVSGVSNNNNNTAVVAERKARPQEKL  NCPRCNSTNTKFCYYNNYSLTQPRYFCKGCRRYWTEGGSLRNVPVGGSSRKNKRSSSSSSSNILQTIPSSLPDLNPPILFSNQIHNKSKGSSQDLNLLSFPVMQDQHHHHVHMSQFLQMPKMEGNGNITHQQQPSSSSSVYGSSSSPVSALELLRTGVNVSSRSGINSSFMPSGSMMDSNTVLYTSSGFPTMVDYKPSNLSFSTDHQGLGHNSNNRSEALHSDHHQQGRVLFPFGDQMKELSSSITQEVDHDDNQQQKSHGNNNNNNNSSPNNGYWSGMFSTTGGGSSW |
| AT3G21270.1 | MQDPAAYYQTMMAKQQQQQQPQFAEQEQLKCPRCDSPNTKFCYYNNYNLSQPRHFCKSCRRYWTKGGALRNVPVGGGSRKNATKRSTSSSSSASSPSNSSQNKKTKNPDPDPDPRNSQKPDLDPTRMLYGFPIGDQDVKGMEIGGSFSSLLANNMQLGLGGGGIMLDGSGWDHPGMGLGLRRTEPGNNNNNPWTDLAMNRAEKN |
| AT3G45610.1 | MDYSSMHQNVMGVSSCSTQDYQNQKKPLSATRPAPPEQSLRCPRCDSTNTKFCYYNNYSLSQPRYFCKSCRRYWTKGGILRNIPIGGAYRKHKRSSSATKSLRTTPEPTMTHDGKSFPTASFGYNNNNISNEQMELGLAYALLNKQPLGVSSHLGFGSSQSPMAMDGVYGTTSHQMENTGYAFGNGGGGMEQMATSDPNRVLWGFPWQMNMGGGSGHGHGHVDQIDSGREIWSSTVNYIN  TGALL |
| AT3G47500.1 | MMMETRDPAIKLFGMKIPFPSVFESAVTVEDDEEDDWSGGDDKSPEKVTPELSDKNNNNCNDNSFNNSKPETLDKEEATSTDQIESSDTPEDNQQTTPDGKTLKKPTKILPCPRCKSMETKFCYYNNYNINQPRHFCKACQRYWTAGGTMRNVPVGAGRRKNKSSSSHYRHITISEALEAARLDPGLQANTRVLSFGLEAQQQHVAAPMTPVMKLQEDQKVSNGARNRFHGLADQRLVARVENGDDCSSGSSVTTSNNHSVDESRAQSGSVVEAQMNNNNNNNMNGYACIPGVPWPYTWNPAMPPPGFYPPPGYPMPFYPYWTIPMLPPHQSSSPISQKCSNTNSPTLGKHPRDEGSSKKDNETERKQKAGCVLVPKTLRIDDPNEAAKSSIWTTLGIKNEAMCKAGGMFKGFDHKTKMYNNDKAENSPVLSANPAALSRSHNFHEQI |
| AT3G50410.1 | MPTSDSGEPRRIAMKPNGVTVPISDQQEQLPCPRCDSSNTKFCYYNNYNFSQPRHFCKACRRYWTHGGTLRDVPVGGGTRKSAKRSRTCSNSSSSSVSGVVSNSNGVPLQTTPVLFPQSSISNGVTHTVTESDGKGSALSLCGSFTSTLLNHNAAATATHGSGSVIGIGGFGIGLGSGFDDVSFGLGRAMWPFSTVGTATTTNVGSNGGHHAVPMPATWQFEGLESNAGGGFVSGEYFAWPDLSITTPGNSLK |
| AT3G52440.1 | MERAEALTSSFIWRPNANANAEITPSCPRCGSSNTKFCYYNNYSLTQPRYFCKGCRRYWTKGGSLRNVPVGGGCRKSRRPKSSSGNNTKTSLTANSGNPGGGSPSIDLALVYANFLNPKPDESILQENCDLATTDFLVDNPTGTSMDPSWSMDINDGHHDHYINPVEHIVEECGYNGLPPFPGEELLSLDTNGVWSDALLIGHNHVDVGVTPVQAVHEPVVHFADESNDSTNLLFGSWSPFDFTADG |
| AT3G55370.1 | MVFSSLPVNQFDSQNWQQQGNQHQLECVTTDQNPNNYLRQLSSPPTSQVAGSSQARVNSMVERARIAKVPLPEAALNCPRCDSTNTKFCYFNNYSLTQPRHFCKTCRRYWTRGGSLRNVPVGGGFRRNKRSKSRSKSTVVVSTDNTTSTSSLTSRPSYSNPSKFHSYGQIPEFNSNLPILPPLQSLGDYNSSNTGLDFGGTQISNMISGMSSSGGILDAWRIPPSQQAQQFPFLINTTGLVQSSNALYPLLEGGVSATQTRNVKAEENDQDRGRDGDGVNNLSRNFLGNININSGRNEEYTSWGGNSSWTGFTSNNSTGHLSF |
| AT3G55370.2 | MVFSSLPVNQFDSQNWQQQGNQHQLECVTTDQNPNNYLRQLSSPPTSQVAGSSQARVNSMVERARIAKVPLPEAALNCPRCDSTNTKFCYFNNYSLTQPRHFCKTCRRYWTRGGSLRNVPVGGGFRRNKRSKSRSKSTVVVSTDNTTSTSSLTSRPSYSNPSKFHSYGQIPEFNSNLPILPPLQSLGDYNSSNTGLDFGGTQISNMISGMSSSGGILDAWRIPPSQQAQQFPFLINTTGLVQSSNALYPLLEGKGGVNQGDSQQKSSDYSNQLMFKPLMDFSSGGVSATQTRNVKAEENDQDRGRDGDGVNNLSRNFLGNININSGRNEEYTSWGGNSSWTGFTSNNSTGHLSF |
| AT3G61850.1 | MDATKWTQGFQEMINVKPMEQMISSTNNNTPQQQPTFIATNTRPNATASNGGSGGNTNNTATMETRKARPQEKVNCPRCNSTNTKFCYYNNYSLTQPRYFCKGCRRYWTEGGSLRNVPVGGSSRKNKRSSTPLASPSNPKLPDLNPPILFSSQIPNKSNKDLNLLSFPVMQDHHHHALELLRSNGVSSRGMNTFLPGQMMDSNSVLYSSLGFPTMPDYKQSNNNLSFSIDHHQGIGHNTINSNQRAQDNNDDMNGASRVLFPFSDMKELSSTTQEKSHGNNTYWNGMFSNTGGSSW |
| AT3G61850.2 | MINVKPMEQMISSTNNNTPQQQPTFIATNTRPNATASNGGSGGNTNNTATMETRKARPQEKVNCPRCNSTNTKFCYYNNYSLTQPRYFCKGCRRYWTEGGSLRNVPVGGSSRKNKRSSTPLASPSNPKLPDLNPPILFSSQIPNKSNKDLNLLSFPVMQDHHHHALELLRSNGVSSRGMNTFLPGQMMDSNSVLYSSLGFPTMPDYKQSNNNLSFSIDHHQGIGHNTINSNQRAQDNNDDMNGASRVLFPFSDMKELSSTTQEKSHGNNTYWNGMFSNTGGSSW |
| AT3G61850.3 | MINVKPMEQMISSTNNNTPQQQPTFIATNTRPNATASNGGSGGNTNNTATMETRKARPQEKVNCPRCNSTNTKFCYYNNYSLTQPRYFCKGCRRYWTEGGSLRNVPVGGSSRKNKRSSTPLASPSNPKLPDLNPPILFSSQIPNKSNKDLNLLSFPVMQDHHHHALELLRSNGVSSRGMNTFLPGQMMDSNSVLYSSLGFPTMPDYKQSNNNLSFSIDHHQGIGHNTINSNQRAQDNNDDMNGASRVLFPFSDMKELSSTTQEKSHGNNTYWNGMFSNTGGSSW |
| AT3G61850.4 | MDATKWTQGFQEMINVKPMEQMISSTNNNTPQQQPTFIATNTRPNATASNGGSGGNTNNTATMETRKARPQEKVNCPRCNSTNTKFCYYNNYSLTQPRYFCKGCRRYWTEGGSLRNVPVGGSSRKNKRSSTPLASPSNPKLPDLNPPILFSSQIPNKSNKDLNLLSFPVMQDHHHHGMSHFFHMPKIENNNTSSSIYASSSPVSALELLRSNGVSSRGMNTFLPGQMMDSNSVLYSSLGFPTMPDYKQSNNNLSFSIDHHQGIGHNTINSNQRAQDNNDDMNGASRVLFPFSDMKELSSTTQEKSHGNNTYWNGMFSNTGGSSW |
| AT4G00940.1 | MDHHQYHHHDQYQHQMMTSTNNNSYNTIVTTQPPPTTTTMDSTTATTMIMDDEKKLMTTMSTRPQEPRNCPRCNSSNTKFCYYNNYSLAQPRYLCKSCRRYWTEGGSLRNVPVGGGSRKNKKLPFPNSSTSSSTKNLPDLNPPFVFTSSASSSNPSKTHQNNNDLSLSFSSPMQDKRAQGHYGHFSEQVVTGGQNCLFQAPMGMIQFRQEYDHEHPKKNLGFSLDRNEEEIGNHDNFVVNEEGSKMMYPYGDHEDRQQHHHVRHDDGNKKREGGSSNELWSGIILGGDSGGPTW |
| AT4G21030.1 | MNNLNVFTNEDNEMNVMPPPRVCPRCYSDQTRFSYFNNNKKSQPRYKCKNCCRCWTHGGVLRNIPVTGICDKSNLPKIDQSSVSQMILAEIQQGNHQPFKKFQENISVSVSSSSDVSIVGNHFDDLSELHGITNSTPIRSFTMDRLDFGEESFQQDLYDVGSNDLIGNPLINQSIGGYVDNHKDEHKLQFEYES |
| AT4G21040.1 | MDNFNVVANEDNQVNDVKPPPPPPRVCARCDSDNTKFCYYNNYSEFQPRYFCKNCRRYWTHGGALRNVPIGGSSRAKRTRINQPSVAQMVSVGIQPGSHKPFFNVQENNDFVGSFGASSSSFVAAVGNRFSSLSHIHGGMVTNVHPTQTFRPNHRLAFHNGSFEQDYYDVGSDNLLVNQQVGGYVDNHNGYHMNQVDQYNWNQSFNNAMNMNYNNASTSGRMHPSHLEKGGP |
| AT4G21050.1 | MDNLNVFANEDNQVNGLKRPPPSRVCPRCDSDNTKFCFYNNYSESQPRYFCKNCRRYWTHGGALRNIPVGGSCRKPKRLKVDQSSISEMVSVENQPINHQSFRQTQENNEFVRSFDASSSATVTAVPNHFGYLSELHGVTNLLPIQSFRTMDCLDFGDESFQQGYYDVGSNDLIDNPLINQSIGGYVDNLTSYCINQVEPKLQPRYEHES |
| AT4G21080.1 | MDNLNVFANEDNQVNDVKPPPPPPRVCARCDSDNTKFCYYNNYCEFQPRYFCKNCRRYWTHGGALRNIPIGGSSRAKRARVNQPSVARMVSVETQRGNNQPFSNVQENVHLVGSFGASSSSSVGAVGNLFGSLYDIHGGMVTNLHPTRTVRPNHRLAFHDGSFEQDYYDVGSDNLLVNQQVGGYGYHMNPVDQFKWNQSFNNTMNMNYNNDSTSGSSRGSDMNVNHDNKKIRYRNSVIMHPCHLEKDGP |
| AT4G24060.1 | MDTAQWPQEIVVKPLEEIVTNTCPKPQPQPLQPQQPPSVGGERKARPEKDQAVNCPRCNSTNTKFCYYNNYSLTQPRYFCKGCRRYWTEGGSLRNIPVGGGSRKNKRSHSSSSDISNNHSDSTQPATKKHLSDHHHHLMSMSQQGLTGQNPKFLETTQQDLNLGFSPHGMIRTNFTDLIHNIGNNTNKSNNNNNPLIVSSCSAMATSSLDLIRNNSNNGNSSNSSFMGFPVHNQDPASGGFSMQDHYKPCNTNTTLLGFSLDHHHNNGFHGGFQGGEEGGEGGDDVNGRHLFPFEDLKLPVSSSSATINVDINEHQKRGSGSDAAATSGGYWTGMLSGGSWC |
| AT4G38000.1 | MMTSSHQSNTTGFKPRRIKTTAKPPRQINNKEPSPATQPVLKCPRCDSVNTKFCYYNNYSLSQPRHYCKNCRRYWTRGGALRNVPIGGSTRNKNKPCSLQVISSPPLFSNGTSSASRELVRNHPSTAMMMMSSGGFSGYMFPLDPNFNLASSSIESLSSFNQDLHQKLQQQRLVTSMFLQDSLPVNEKTVMFQNVELIPPSTVTTDWVFDRFATGGGATSGNHEDNDDGEGNLGNWFHNANNNALL |
| AT5G02460.1 | MVFSSFPTYPDHSSNWQQQHQPITTTVGFTGNNINQQFLPHHPLPPQQQQTPPQLHHNNGNGGVAVPGGPGGLIRPGSMAERARLANIPLPETALKCPRCDSTNTKFCYFNNYSLTQPRHFCKACRRYWTRGGALRSVPVGGGCRRNKRTKNSSGGGGGSTSSGNSKSQDSATSNDQYHHRAMANNQMGPPSSSSSLSSLLSSYNAGLIPGHDHNSNNNNILGLGSSLPPLKLMPPLDFTDNFTLQYGAVSAPSYHIGGGSSGGAAALLNGFDQWRFPATNQLPLGGLDPFDQQHQMEQQNPGYGLVTGSGQYRPKNIFHNLISSSSSASSAMVTATASQLASVKMEDSNNQLNLSRQLFGDEQQLWNIHGAAAASTAAATSSWSEVSNNFSSSSTSNI |
| AT5G39660.1 | MADPAIKLFGKTIPLPELGVVDSSSSYTGFLTETQIPVRLSDSCTGDDDDEEMGDSGLGREEGDDVGDGGGESETDKKEEKDSECQEESLRNESNDVTTTTSGITEKTETTKAAKTNEESGGTACSQEGKLKKPDKILPCPRCNSMETKFCYYNNYNVNQPRHFCKKCQRYWTAGGTMRNVPVGAGRRKNKSPASHYNRHVSITSAEAMQKVARTDLQHPNGANLLTFGSDSVLCESMASGLNLVEKSLLKTQTVLQEPNEGLKITVPLNQTNEEAGTVSPLPKVPCFPGPPPTWPYAWNGVSWTILPFYPPPAYWSCPGVSPGAWNSFTWMPQPNSPSGSNPNSPTLGKHSRDENAAEPGTAFDETESLGREKSKPERCLWVPKTLRIDDPEEAAKSSIWETLGIKKDENADTFGAFRSSTKEKSSLSEGRLPGRRPELQANPAALSRSANFHESS |
| AT5G39660.2 | MADPAIKLFGKTIPLPELGVVDSSSSYTGFLTETQIPVRLSDSCTGDDDDEEMGDSGLGREEGDDVGDGGGESETDKKEEKDSECQEESLRNESNDVTTTTSGITEKTETTKAAKTNEESGGTACSQEGKLKKPDKILPCPRCNSMETKFCYYNNYNVNQPRHFCKKCQRYWTAGGTMRNVPVGAGRRKNKSPASHYNRHVSITSAEAMQKVARTDLQHPNGANLLTFGSDSVLCESMASGLNLVEKSLLKTQTVLQEPNEGLKITVPLNQTNEEAGTVSPLPKVPCFPGPPPTWPYAWNGVSWTILPFYPPPAYWSCPGVSPGAWNSFTWMPQPNSPSGSNPNSPTLGKHSRDENAAEPGTAFDETESLGREKSKPERCLWVPKTLRIDDPEEAAKSSIWETLGIKKDENADTFGAFRSSTKEKSSLSEGRLPGRRPELQANPAALSRSANFHESS |
| AT5G60200.1 | MDHLLQHQDVFGNYNKAREAMGLSYSSNPTPLDNDQKKPSPATAVTRPQPPELALRCPRCDSTNTKFCYYNNYSLTQPRYFCKSCRRYWTKGGTLRNIPVGGGCRKNKRSTSSAARSLRTTPEPASHDGKVFSAAGFNGYSNNEHIDLSLAFALLNKQHPGSSSQLGFHSELGSSHQSDMEGMFGTSQQKENATYAFGNGSSGLGDPSRVLWGFPWQMNGESFGMMNIGGGGGHVDQIDSGREMWTNMNYINSGALM |
| AT5G60850.1 | MQDIHDFSMNGVGGGGGGGGRFFGGGIGGGGGGDRRMRAHQNNILNHHQSLKCPRCNSLNTKFCYYNNYNLSQPRHFCKNCRRYWTKGGVLRNVPVGGGCRKAKRSKTKQVPSSSSADKPTTTQDDHHVEEKSSTGSHSSSESSSLTASNSTTVAAVSVTAAAEVASSVIPGFDMPNMKIYGNGIEWSTLLGQGSSAGGVFSEIGGFPAVSAIETTPFGFGGKFVNQDDHLKLEGETVQQQQFGDRTAQVEFQGRSSDPNMGFEPLDWGSGGGDQTLFDLTSTVDHAYWSQSQWTSSDQDQSGLYLP |
| AT5G62430.1 | MLETKDPAIKLFGMKIPFPTVLEVADEEEEKNQNKTLTDQSEKDKTLKKPTKILPCPRCNSMETKFCYYNNYNVNQPRHFCKACQRYWTSGGTMRSVPIGAGRRKNKNNSPTSHYHHVTISETNGPVLSFSLGDDQKVSSNRFGNQKLVARIENNDERSNNNTSNGLNCFPGVSWPYTWNPAFYPVYPYWSMPVLSSPVSSSPTSTLGKHSRDEDETVKQKQRNGSVLVPKTLRIDDPNEAAKSSIWTTLGIKNEVMFNGFGSKKEVKLSNKEETETSLVLCANPAALSRSINFHEQM |
| AT5G62940.1 | MGLTSLQVCMDSDWLQESESSGGSMLDSSTNSPSAADILAACSTRPQASAVAVAAAALMDGGRRLRPPHDHPQKCPRCESTHTKFCYYNNYSLSQPRYFCKTCRRYWTKGGTLRNIPVGGGCRKNKKPSSSNSSSSTSSGKKPSNIVTANTSDLMALAHSHQNYQHSPLGFSHFGGMMGSYSTPEHGNVGFLESKYGGLLSQSPRPIDFLDSKFDLMGVNNDNLVMVNHGSNGDHHHHHNHHMGLNHGVGLNNNNNNGGFNGISTGGNGNGGGLMDISTCQRLMLSNYDHHHYNHQEDHQRVATIMDVKPNPKLLSLDWQ  QDQCYSNGGGSGGAGKSDGGGYGNGGYINGLGSSWNGLMNGYGTSTKTNSLV |
| AT5G65590.1 | MSSHTNLPSPKPVPKPDHRISGTSQTKKPPSSSVAQDQQNLKCPRCNSPNTKFCYYNNYSLSQPRHFCKSCRRYWTRGGALRNVPIGGGCRKTKKSIKPNSSMNTLPSSSSSQRFFSSIMEDSSKFFPPPTTMDFQLAGLSLNKMNDLQLLNNQEVLDLRPMMSSGRENTPVDVGSGLSLMGFGDFNNNHSPTGFTTAGASDGNLASSIETLSCLNQDLHWRLQQQRMAMLFGNSKEETVVVERPQPILYRNLEIVNSSSPSSPTKKGDNQTEWYFGNNSDNEGVISNNANTGGGGSEWNNGIQAWTDLNHYNALP |
| AT5G66940.1 | MPSEFSESRRVPKIPHGQGGSVAIPTDQQEQLSCPRCESTNTKFCYYNNYNFSQPRHFCKSCRRYWTHGGTLRDIPVGGVSRKSSKRSRTYSSAATTSVVGSRNFPLQATPVLFPQSSSNGGITTAKGSASSFYGGFSSLINYNAAVSRNGPGGGFNGPDAFGLGLGHGSYYEDVRYGQGITVWPFSSGATDAATTTSHIAQIPATWQFEGQESKVGFVSGDYVA |
